# Importance of test–retest reliability for promoting fMRI based screening and interventions in major depressive disorder

**DOI:** 10.1038/s41398-021-01507-3

**Published:** 2021-07-10

**Authors:** Laurie Compère, Greg J. Siegle, Kymberly Young

**Affiliations:** Department of Psychiatry, University of Pittsburgh School of Medicine, Western Psychiatric Institute and Clinic, Pittsburgh, PA USA

**Keywords:** Depression, Neuroscience

## Abstract

Proponents of personalized medicine have promoted neuroimaging in three areas of clinical application for major depression: clinical prediction, outcome evaluation, and treatment, via neurofeedback. Whereas psychometric considerations such as test–retest reliability are basic precursors to clinical adoption for most clinical instruments, we show, in this article, that basic psychometrics have not been regularly attended to in fMRI of depression. For instance, no fMRI neurofeedback study has included measures of test–retest reliability, despite the implicit assumption that brain signals are stable enough to train. We consider several factors that could be useful to aid clinical translation, including (1) attending to how the BOLD response is parameterized, (2) identifying and promoting regions or voxels with stronger psychometric properties, (3) accounting for within-individual changes (e.g., in symptomatology) across time, and (4) focusing on tasks and clinical populations that are relevant for the intended clinical application. We apply these principles to published prognostic and neurofeedback data sets. The broad implication of this work is that attention to psychometrics is important for clinical adoption of mechanistic assessment, is feasible, and may improve the underlying science.

## Introduction

The idea that fMRI could have therapeutic utility is based on assumptions that hemodynamic activity is reliable over time in the absence of intervention, and that observed changes between one scan session and the next to have significant and interpretable values [1] and though best practice guidelines are emerging [2, 3], they are not focused on clinical applications. Here, we consider the state of and possible ways to improve test–retest reliability in task-based fMRI biomarker and neurofeedback (fMRI-nf) designs for clinical psychiatric applications, using major depressive disorder (MDD) as a running case example. We demonstrate our suggested principles on published MDD neuroimaging biomarker treatment outcome and neurofeedback datasets as proofs of concept. Reliability in clinical applications of fMRI is particularly important as it is assumed for understanding recovery and change processes [4].

To index test–retest reliability, we consider the standard index in fMRI, the intraclass correlation (ICC) [5]. The ICC reflects rank ordering of values across days [6]. Values range from 0 (no reliability) to 1 (perfect reliability), where values of less than 0.4 are often considered poor, 0.4–0.59 fair, 0.60–0.74 good, and above 0.75 excellent [7–9], though more stringent cutoffs have also been recommended [10]. ICC-based reliability estimates have been rarely reported in fMRI studies and usually reveal poor reliability when estimated [1]. Non-clinical studies have generally found low to moderate test–retest reliability values for regional fMRI activity, with ICCs ranging from 0.33–0.66 [6].

Of various classes of ICC [7], the most frequently employed is ICC (3, 1) which assumes variance is common across scanners. To best match the literature and to investigate the impact of taking into account clinical and design covariates (i.e., including scanner) when computing reliability indexes, we focus then on this one. Though some popular packages (SPM, FSL) do not inherently support computation of this metric; add-on packages (e.g., “reliability toolbox” for SPM) do allow such computations (see Supplementary Section 1).

### Biomarker treatment outcome studies review

Many studies use fMRI to predict treatment outcomes in MDD [11–14]. We surveyed this literature to examine whether reliability has been considered and is adequate for clinical application.

### Method

A PubMed search with the keywords “fMRI AND biomarker OR prediction OR predict AND depression OR MDD OR major depressive disorder NOT Rest NOT Resting” produced 140,640 results in December 2018. We combined this list with other articles discovered in our submitted fMRI meta-analysis of depression treatment outcome prediction studies [15] to complete the list of articles. After removing articles, not including functional neuroimaging (i.e., studies focusing on volumetric measures or using PET) or human participants, we were left with 55 studies (Supplementary Section 2).

### Results

Though most of the reviewed studies could have reported test–retest reliability (i.e., participants performed two scans), most did not mention it. Seven mentioned reliability in the discussion and only one reported test–retest reliability at the subject level [16]. Other studies that mention reliability describe the stability of group effects, which does not reflect test–retest reliability at the individual subject level.

### rtfMRI-nf studies review

Interventions that use biological measures as real-time targets, including rtfMRI-nf, which trains patients to regulate the hemodynamic activity in regions of interest, also assume reliability. Thus, we considered whether test–retest reliability is being reported in the fMRI neurofeedback literature.

### Method

A PubMed search with the key words “(neurofeedback AND fMRI) OR (rt-fMRI-nf) AND (depression OR MDD OR major depressive disorder)” provided 44 results in December 2018. After removing articles, not including rtfMRI-nf or patients suffering from MDD, we were left with 11 studies (Supplementary Section 3).

### Results

None of the examined fMRI-nf studies reported on the reliability of the signal being trained (Supplementary Section 3 and 4 for specific discussion of functional localizers).

### Conclusions thus far

MDD studies using fMRI for clinical prediction or treatment rarely mention reliability.

## Optimizing test–retest reliability in fMRI/rtfMRI-nf

One possible reason test-reliability is rarely considered is that it is too low to state without shame (with reported ICC’s for fMRI studies generally in the ~0.50 region, which is below usual “good” reliability thresholds). Thus, the remainder of this article is dedicated to introducing ways to report, improve, and increase the clinical applicability of test–retest reliability for fMRI. We apply and evaluate these suggestions in published fMRI depression data sets [17, 18]. Optimizing preprocessing is already well known to increase the measurement of true signal, and thus reliability [19–22]. We, therefore, begin by considering whether using alternate ways of indexing task-related reactivity in single-subject data lead to further improvements.

### R1) Optimize indices of task-related reactivity

Mis-specification of the shape of BOLD reactivity can introduce inefficiency and noise into model-based task-reactivity estimates, which decreases reliability [23–25], e.g., by not accounting for systematically sustained neural responses to task stimuli in a clinical population such as depression (e.g., [26]). Thus, we propose evaluating indices such as the average amplitude and timing/shape of the curve of the BOLD response in addition to its canonical amplitude. Gamma variate models, in particular, yield parameters for onset, rise and fall slopes, and magnitude of hemodynamic responses. Similarly, including temporal and dispersion derivatives can account for individual differences in peak response timing and small differences in HRF length, providing larger test–retest reliability values [27].

### R2) Examine voxel-wise reliability within regions of interest (ROIs)

Caceres et al. (2009) [5] suggest reporting the median of voxelwise ICC’s within a ROI to index its test–retest reliability [27, 28]. This approach assumes heterogeneity within the ROI, which may not always be the case [29]. Just as questionnaires are traditionally constructed by eliminating unreliable items from an initial theoretically plausible set [30], an index that inherits solely from the reliable voxels may increase the psychometric properties of preserved portions of regions.

### R3) Optimize models to account for individual and clinical features

Minimizing sources of non-interest that could vary between administrations increases the reliability of acquired data [31]. Some time-varying noise sources such as differences in instrumentation, time of day, motion, instructions, practice, and training effects, can be controlled via design [4, 32]. Other sources of variance may be controlled statistically, e.g., clinical features such as state anxiety and rumination which can account for neural activation [26, 33, 34] and habituation [35].

### R4) Examine reliability within relevant clinical populations

The majority of fMRI studies that report reliability have recruited healthy, often young, university students [6, 28], which does not account for the idea that paradigms that address clinical phenomenology may be reliable in individuals with clinical features but not controls. As groups might differ in the degree to which regional signals are reliable between measurements [27], and because ICCs are proportional to between-subject variability, heterogeneous samples can produce different ICCs even with the same degree of within-subject reliability of test–retest values. Thus, testing reliability in the population (e.g., treatment-seeking patients), task, and regions of interest may provide more accurate estimates.

## Evaluation of suggested optimizations in a prognostic neuroimaging treatment outcome dataset

In this section we demonstrate feasibility of R1-R4 and examine whether they are useful when applied to a published clinical fMRI depression dataset [18]. Our code is available from https://github.com/PICANlab/Reliability_toolbox in the “activation_task_reliability” folder.

### Method

The Siegle et al. (2012) [18] is a MDD neuroimaging treatment outcome which sample was augmented by *N* = 8 who completed the protocol after that paper’s submission, yielding 57 patients with MDD, and 35 healthy control participants (see Supplementary Section 5 for details and sample relationship to Siegle et al 2012). Briefly, participants completed a slow event-related task during 3T fMRI in which they labeled the valence of emotional words (here, as in the published dataset, we analyzed only nominally negative words) before and after 12–16 weeks of Cognitive Therapy for patients with MDD while the control group received no intervention.

We computed reliability estimates within four ROIs which the literature suggests may function as biomarkers for treatment response including the amygdala [36–38], dorsolateral prefrontal cortex (DLPFC [39]), rostral anterior cingulate cortex (rACC [40]) and subgenual cingulate cortex (sgACC [16, 41, 42], region-wise definitions in Supplementary Section 6).

### Optimize the BOLD Signal

The BOLD response to negative words was modeled within participants using four methods including (1) amplitude of a canonically shaped BOLD signal using AFNI’s 3dDeconvolve with a narrow tent function (‘BLOCK5(1,1)‘ [43]), (2) Area under the curve (via multiple regression of a delta function across eight TRs using 3dDeconvolve, i.e., computed with Finite Impulse Response/FIR basis, with sum of betas as the parameter retained), (3) Peak amplitude from the same regressions as #2, and (4) a gamma variate model with parameters for onset-delay, rise–decay rate, and height. Voxelwise outliers outside the Tukey hinges were Windsorized across participants and ICCs (3,1) were computed [7] within individuals for each modeling method using custom Matlab code. While ICC (2,1) allows generalizing results obtained from different scanners, we chose to use ICC (3,1) to be able to compare with most of the literature, given that it is the most widely used ICC. This approach also allowed us to examine the importance of including scanner as a covariate in 3.1.3.

### Compute voxelwise reliability

To measure the benefit of identifying reliable voxels, we calculated the mean, median, and standard deviation of the ICCs throughout each of the ROIs for each modeling method and each group.

### Include clinical and design-related measures

We examined whether indices of reliability increased when clinical and design-related measures were included. As the ICC does not easily allow the inclusion of covariates, we used semi partial correlations within the context of multiple regressions with and without covariates to assess changes in reliability, where covariates were pre and post clinical measures, as:

Post = β0 + β(1→n)covariates + β(n + 1Pre)

This model accounts for the potential that participants who show little change in symptoms may have better test–retest reliability. Modeling these clinical effects at the group level should make it possible to identify variance unique to test–retest reliability.

We included indices of pre- and post-treatment depressive symptomatology (Beck Depression Inventory; BDI [44]), state and trait anxiety [45], rumination [46], and sleepiness [47] administered on the scan day, the scanner on which data were acquired, and participant’s group when patients and controls were considered in one sample, coded as dummy variables, as covariates. Missing data were imputed via regression from the other administered measures also used as covariates.

A primary question was whether any of R1-4, would differentially affect reliability estimates. As such, after computing reliability estimates at each voxel, we rank ordered them across all permutations of BOLD estimate parameters (six parameters) and the use or non-use of covariates (two conditions) at each voxel per ROI, yielding 12 x #-voxels rankings per ROI. Following a Kolgomorov–Smirnov test justifying the need to use non-parametric tests, we report a Kruskal-Wallis test to determine whether the rankings differed across models in each ROI. If they did, as a simple effects test, we generated confidence intervals around the mean of rankings for each of the 12 conditions via a one-way ANOVA (via Matlab’s multcompare function). Non-overlapping confidence intervals are interpretable as significant differences between one condition and any other. To display them we generated figures showing the mean of rankings for each condition, which will be numbers on the order of one to 12 x #-voxels, with higher means representing being at the top of the rankings across many voxels within the ROI.

### Use clinically representative samples

All analyses were conducted on the whole sample (controls and patients) to establish likely reliability of tests that could be used to discriminate groups, and on patients only, to establish likely reliability of clinical prognostic and change indicators. We considered multiple reliability effect size thresholds which might be used in other studies (0.4 and 0.6 for fair and good reliability and 0.7, and 0.75 for traditional labels of the data as “reliable” and clinically meaningful).

### Type 1 error control

As (1) each of the hypotheses and regions examined for this manuscript was considered a different family of tests and (2) we want our results to generalize to reliability as it is reported in the confirmatory biomarker and neurofeedback literature where only one region is generally examined, consistent with the literature on test–retest reliability in neuroimaging, type I error was not controlled across regions and hypotheses for ROI-wise statistics. For simple-effects tests of differences in rankings across conditions, we controlled for the number of conditions with a Bonferroni test. For voxelwise statistics, we subjected all voxelwise residual maps to empirical cluster thresholding (AFNI’s 3dFWHMx and 3dClustSim, acf model with small-volume corrections for examined regions) using a p threshold (-pthr) based on each considered effect size threshold (see Supplementary Section 7 for more details).

### Results and discussion

#### Optimizing the BOLD signal

**Fig. 1:**
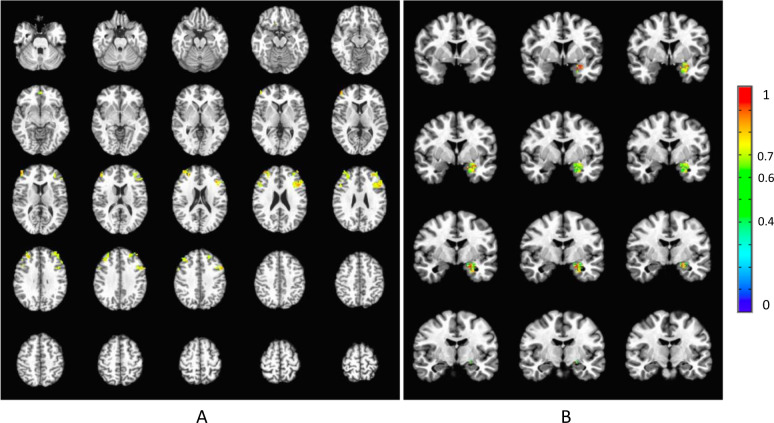
Test-retest reliability in ROIs estimated with voxel wise ICCs using height parameter. **A** threshold of ICC > 0.4 and cluster correction areapplied for this threshold. In panel **A.**, the results are represented for the Siegle et al. (2012) dataset of patients and in panel **B**., the results are represented for Young et al. (2017) data set of the transfer run in the experimental group (signal with training) preprocessed with the TBV style pipeline.

ICC’s were uniformly low (<0.3) for all BOLD parameterizations when entire ROIs were considered (Table 1). Kruskal–Wallis tests did suggest differential reliability across our parameterizations (Supplementary Section 8). This held when the two outlying uniformly low-reliability parameterizations (rise decay with and without covariates) were removed from consideration (Supplementary Section 9). Yet, there were non-overlapping confidence intervals among counts of rank orderings of parameterizations for voxelwise tests, suggesting that at least for some subsets of regions, some parameterizations were superior (Supplementary Section 10 and 11). For example, in the full sample, for the amygdala, amplitude without covariates was superior to other parameters. Overall ROIs, the most reliable parameters were amplitude, canonical amplitude, and height (Fig. 1A shows voxelwise variation within a priori ROIs for the height parameter) for the whole sample and amplitude, the area under the curve, and height for only patients (Supplementary Section 10 and 11). However, looking at ROIs and samples independently, the parameter offering the highest levels of reliability varied.

**Table 1:** Table of mean, standard deviation and median values of ICCs for each sample, reactivity model, and ROI.

Population		Reactivity model	Amygdala	DLPFC	rACC	sgACC liberally thresholded	sgACC conservatively thresholded
Controls & patients		Canonical amplitude	0.11 (±0.09); 0.11	0.24 (±0.16); 0.26	0.09 (±0.10); 0.09	0.15 (±0.08); 0.13	0.17 (±0.09); 0.18
		Amplitude	0.23 (±0.14); 0.22	0.12 (±0.11); 0.12	0.11 (±0.10); 0.12	−0.01 (±0.13); −0.04	−0.04 (±0.14); −0.08
		Area under the curve	0.13 (±0.14); 0.12	0.08 (±0.10); 0.07	0.03 (±0.11); 0.03	−0.03 (±0.09); −0.04	−0.06 (±0.10); −0.07
		Onset delay	0 (±0.09); −0.01	0.01 (±0.09); 0	0 (±0.10); 0	0 (±0.08); 0.01	−0.01 (±0.10); 0
		Rise decay	0 (±0); 0	0 (±0); 0	0 (±0); 0	0 (±0); 0	0 (±0); 0
		Height	0.08 (±0.10); 0.09	0.21 (±0.15); 0.23	0.13 (±0.12); 0.14	0.16 (±0.12); 0.17	0.18 (±0.12); 0.23
Patients		Canonical amplitude	0.09 (±0.11); 0.11	0.22 (±0.16); 0.23	0.08 (±0.14); 0.08	0.10 (±0.12); 0.07	0.14 (±0.15); 0.12
		Amplitude	0.22 (±0.15); 0.22	0.11 (±0.13); 0.11	0.10 (±0.13); 0.11	−0.06 (±0.15); −0.07	−0.08 (±0.14); −0.08
		Area under the curve	0.13 (±0.14); 0.12	0.6 (±0.12); 0.03	0.03 (±0.13); 0.04	−0.08 (±0.13); −0.08	−0.10 (±0.13); −0.09
		Onset delay	−0.01 (±0.12); −0.01	0.01 (±0.12); 0	−0.01 (±0.13); −0.01	0.02 (±0.11); 0.02	0.01 (±0.12); 0.05
		Rise decay	0 (±0); 0	0 (±0); 0	0 (±0); 0	0 (±0); 0	0 (±0); 0
		Height	0.09 (±0.12); 0.08	0.22 (±0.16); 0.23	0.12 (±0.15); 0.13	0.16 (±0.17); 0.18	0.17 (±0.17); 0.21

Mean (±standard deviation); median

#### Voxelwise reliability

In the whole sample, moderate reliability (ICC > 0.4) in clusters large enough to infer significance was observed in the DLPFC using the canonical amplitude model and in the amygdala using amplitude (Table 2). “Good” (ICC > 0.6) reliability was reached in clusters large enough to infer significance when only the patients were considered, using amplitude and height in the DLPFC. These levels of voxelwise test–retest reliability were higher than using the median or mean value of ICCs within whole ROIs (Table 1). Levels of generally accepted reliability for clinical measures (ICC > 0.7) were not observed in clusters large enough to report.

**Table 2:** Table of number of voxels reaching different reliability thresholds for each sample, first level parameter, and ROI with cluster correction applied.

ROI		Amydgala, (242 voxels)		DLPFC, (2675 voxels)		rACC (865 voxels)		sgACC liberally thresholded, (33 voxels)		sgACC conservatively thresholded, (18 voxels)	
		ICC thresholds		ICC thresholds		ICC thresholds		ICC thresholds		ICC thresholds	
Population	Reactivity model	0.4	0.6	0.4	0.6	0.4	0.6	0.4	0.6	0.4	0.6
Controls & patients	Canonical amplitude	0	0	465	0	0	0	0	0	0	0
	Amplitude	66	0	5	0	0	0	0	0	0	0
	Area under the curve	10	0	0	0	0	0	0	0	0	0
	Onset delay	0	0	0	0	0	0	0	0	0	0
	Rise decay	0	0	0	0	0	0	0	0	0	0
	Height	0	0	290	2	0	0	0	0	0	0
Patients	Canonical amplitude	0	0	299	6	6	0	0	0	0	0
	Amplitude	24	0	0	0	0	0	0	0	0	0
	Area under the curve	0	0	0	0	0	0	0	0	0	0
	Onset delay	0	0	0	0	0	0	0	0	0	0
	Rise decay	0	0	0	0	0	0	0	0	0	0
	Height	0	0	374	5	5	0	2	0	1	0

#### Clinical and design-related measures

The addition of covariates never resulted in significantly higher average ranks for semi partial correlations in any ROI, in the whole sample or just the patients (Supplementary Section 10). In other words, adding covariates did not improve the reliability, and in some instances made it worse.

## Evaluation of suggested optimizations in an empirical neurofeedback dataset

To further support the feasibility of applying these recommendations and to evaluate the consistency of their performance in a second dataset, we consider a published fMRI neurofeedback dataset [17].

### Method

This dataset constituted 18 patients in the experimental group who received amygdala neurofeedback and 16 patients in the control group who received parietal neurofeedback. Briefly, participants completed two training scans on different days within two weeks, each including a “baseline” and “transfer” runs during which no feedback was presented. The analyzed task was a 40-s per block design during which participants alternately rested, worked to upregulate a target region during recall of positive memories, and did a distraction (counting) task (see Supplementary Section 5 for details of this dataset). Here, we focus on (a) the baseline data on the two training days in control-feedback participants during recall of positive autobiographical memories prior to neurofeedback training. As their amygdala signal did not change over the course of the study at the group level [17], this allows us to examine test–retest reliability of the left amygdala signal without the influence of neurofeedback. (b) the left amygdala signal during the two transfer runs in the experimental group, as this represents the effect of neurofeedback training. Activity during the two post-training transfer runs did not differ at the group level, allowing us to examine the test–retest reliability of the amygdala signal after neurofeedback training. Because this dataset only included patients with MDD, only R1-3 are evaluated in this dataset.

#### Feedback signal

To analyze the feedback signal averaged over the left amygdala we used the output of the script used in [17] that allowed computation of the feedback signal in real-time before considering the voxel-wise signal.

#### Voxel-wise

As rtfMRI-nf involves real-time preprocessing of the data, we sought to examine whether this kind of preprocessing could affect the test–retest reliability of the signal. We, therefore, performed data preprocessing emulating the real-time data processing performed by the commercially available neurofeedback software Turbo BrainVoyager (BrainVoyager, The Netherlands; henceforth “TBV style”) and a more classic contemporary post-hoc preprocessing stream (here referred to as “standard preprocessing”). Both streams were implemented using AFNI.

### TBV style preprocessing

Turbo BrainVoyager performs the following functions in real-time: 3D motion correction, spatial smoothing, and drift removal via the design matrix. We used AFNI to approximate these steps. After spatially transforming the anatomical then functionals to the International Consortium for Brain Mapping 152 template, we then rescaled them to conform to the Talairach atlas dimensions and then performed motion correction to the first image, spatial smoothing 4mm FWHM smoothing kernel and fourth-order detrend for drift removal.

### Standard preprocessing

MRI pre-processing included despiking, volume registration, and slice timing correction for all EPI volumes in a given exam. After applying an intensity uniformity correction on the anatomical, the anatomical was spatially transformed to the International Consortium for Brain Mapping (ICBM) 152 template and rescaled to conform to the Talairach atlas dimensions. Then, the fMRI data for each run were warped nonlinearly and the same spatial transformations were applied. The fMRI run was spatially smoothed within the gray matter mask using a Gaussian kernel with full width at half maximum (FWHM) of 4 mm. GLM analysis was then applied separately for each of the fMRI runs to derive contrasts. The following regressors were included in the GLM model: six motion parameters and their derivatives as nuisance covariates to account for artifacts caused by head motion, white matter, and cerebrospinal fluid signals, and five polynomial terms for modeling drift.

### Optimize the BOLD signal

#### Amygdala signal

From each participant’s real-time left amygdala signal we calculated an “amygdala signal” for each positive recall block minus the mean of the preceding rest block from the output of previously used scripts for real-time preprocessing [17], and recreated the feedback signal by taking the amount of activation at every TR during the experimental condition minus the mean activation in the previous rest condition, on the baseline run of control participants at visits 1 and 2 (signal without training) and on the transfer run of experimental participants at visits 1 and 2 (signal with training), independently. We then averaged the time course of the feedback signal over all upregulate blocks during which neurofeedback was provided. We summarized the activation for each participant for each visit by either a mean of the amygdala signal or by fitting the time course with a gamma variate model with parameters for onset-delay, rise-decay-rate, and height (see Supplementary Section 12 for more information of this methodological choice). ICC (3, 1) estimates were computed [7] independently on the estimates of the feedback signal with and without training.

### Voxelwise signal

The same reactivity models as in the treatment outcome dataset were applied (see section 3.2.1) to data preprocessed with both types of preprocessing but adapted to this design (AFNI tent parameters to accommodate 40 s blocks as BLOCK(40, 1), and area under the curve across entire blocks).

### Compute voxelwise reliability

As in the treatment outcome data set, to measure the benefit of identifying reliable voxels, we calculated the mean, median, and standard deviation of the ICCs in the left amygdala for each model, group, and additionally for both preprocessing pipelines.

### Include clinical and design-related measures

As in the treatment outcome data set, semi partial correlations were computed with and without covariates. We included indices of depressive symptomatology (Beck Depression Inventory; BDI [44]), state and trait anxiety [45], sleepiness and drowsiness administered on the scan day, and the scanner on which data were acquired coded as dummy variables, as covariates. There was no missing data. We then compared the semi-partial correlations across all models of individual responses with and without covariates for each group and preprocessing pipeline as in section 3.1.3, to understand which models offered adequate test–retest reliability and whether there were differences between them.

### Type 1 error control

As discussed in section 3.1.5, cluster correction was applied on voxelwise statistics (details in Supplementary Section 13).

## Results and discussion

### Optimizing the BOLD signal

#### Amygdala signal

The mean amygdala signals with and without training showed poor reliability (ICCs < 0.1). When the signal within the left amygdala was fit using a gamma variate function, the onset-delay and height parameters showed fair reliability for the signal without training (ICC = 0.54 and ICC = 0.47, respectively), with all other models, including those with training, showing minimal reliability (ICC < 0.1). Therefore, it appears that the shape of the signal without training is consistent across sessions and that the signal in the left amygdala is more reliable when unchanged by training, which is consistent with the assumption that training is changing the signal over time.

#### Voxel-wise signal

Kruskal Wallis tests suggested there were differences between the parameters in reliability (Supplementary Sections 14 and 15). In particular, reliability for the height parameter (as well as amplitude for the signal without training) was higher than for other parameters (Supplementary Section 10). The height parameter also yielded a large enough cluster to infer significance for “excellent” (ICC > 0.7) reliability in both samples (Table 3, Fig. 1 for illustration).

**Table 3:** Table of number of voxels reaching different reliability thresholds for each sample, preprocessing, and first-level parameter with cluster correction applied.

ROI		Amygdala (214 voxels)							
Preprocessing		BV style				Standard			
		ICC thresholds				ICC thresholds			
Population	First level model	0.4	0.6	0.7	0.75	0.4	0.6	0.7	0.75
Without training–control–baseline	Canonical amplitude	0	0	0	0	0	0	0	0
	Amplitude	52	16	6	2	35	0	0	0
	Area under the curve	0	0	0	0	40	0	0	0
	Onset-delay	0	0	0	0	0	0	0	0
	Rise-decay	0	0	0	0	0	0	0	0
	Height	78	26	13	13	53	24	9	5
With training–experimental–transfer	Canonical amplitude	0	0	0	0	0	0	0	0
	Amplitude	66	4	2	2	42	11	3	2
	Area under the curve	0	0	0	0	0	0	0	0
	Onset-delay	0	4	4	4	0	5	5	5
	Rise-decay	0	0	0	0	0	0	0	0
	Height	159	81	25	16	73	47	24	21

The use of the standard preprocessing stream had non-significantly different reliabilities from the stream emulating the real-time preprocessing run by Turbo BrainVoyager over all parameters with or without covariates, with the exception of the height parameter without covariates, which showed higher reliability with TBV style preprocessing than with standard preprocessing in the signal without training (Supplementary Section 10).

#### Voxelwise reliability

Some voxelwise ICC values obtained were higher than those computed on the real-time signal covering the entire left amygdala or mean or median ICC values computed over the entire left amygdala (Table 3 vs statistics reported in 4.2.1.1 and Table 4), with some clusters achieving an excellent level of reliability (ICC > 0.7, see Table 3) for standard and TBV-like preprocessing both for the trained and untrained signals, which did not occur for the region as a whole.

**Table 4:** Table of mean, standard deviation, and median values of ICCs for each sample, preprocessing, and first-level parameter with cluster correction applied.

Preprocessing		TBV style	Standard
Without training–control–baseline	Canonical amplitude	−0.07 (±0.21); −0.09	0.01 (±0.24); 0
	Amplitude	0.29 (±0.2); 0.3	0.26 (±0.22); 0.27
	Area under the curve	0.02 (±0.21); 0.01	0.21 (±0.23); 0.18
	Onset-delay	−0.03 (±0.23); −0.05	−0.11 (±0.20); −0.14
	Rise-decay	NA (±NA); NA	NA (±NA); NA
	Height	0.36 (±0.23); 0.33	0.17 (±0.38); 0.24
With training–experimental–transfer	Canonical amplitude	−0.11 (±0.21); −0.12	0.08 (±0.21); 0.09
	Amplitude	0.3 (±0.18); 0.31	0.26 (±0.21); 0.25
	Area under the curve	0.06 (±0.20); 0.07	0.13 (±0.18); 0.13
	Onset-delay	0.02 (±0.24); −0.02	−0.05 (±0.24); −0.13
	Rise-decay	NA (±NA); NA	NA (±NA); NA
	Height	0.52 (±0.19); 0.56	0.35 (±0.28); 0.34

Mean (±standard deviation); median

### Clinical and design-related measures

#### Amygdala signal

Adding covariates when computing semi-partial correlations over the mean amygdala signal improved reliability estimates for the signal without training (mean: from sr = 0.06 to sr = 0.12, with AIC = −90.35 to AIC = −115.21, onset-delay: from sr = 0.14 to sr = 0.21, with AIC = 149.78 to AIC = 139.34, rise-decay: from sr = 0.03 to sr = 0.14, with AIC = 121.91 to AIC = 42.67, height: from sr = 0.16 to sr = 0.29, with AIC = −46.91 to AIC = −63.49) although in no case did we achieve a fair level of reliability (sr < 0.4).

#### Voxelwise signal

The addition of covariates in never resulted in higher average ranks of semipartial correlation distributions on the untrained or trained signal preprocessed with the TBV-like or standard pipeline (Supplementary Section 10).

## Discussion

As stated in a recent meta-analysis [1], task fMRI reliability is not systematically evaluated and when it is, task-related fMRI measures show poor reliability. However, as stated by a comment in response to this meta-analysis [48], we believe that fMRI can have significant test–retest reliability when the right measures are used. Our literature review shows that both prognostic and interventional fMRI studies in MDD, which might otherwise be poised for clinical translation, also do not attend to reliability. Although these results should be replicated in an independent sample, we demonstrate that attending to some fairly simple principles appears to improve reliability in the examined datasets (Fig. 1). These principles include careful modeling of the BOLD signal, identification of reliable voxels within regions of interest, and calculation of reliability in the population for which translational applications are being considered. Across both datasets, the height parameter from a gamma variate function was the most reliable way to model the BOLD signal, especially among patients with MDD, in some regions of interest, and was, in some combinations of region and population or training condition, more reliable than the canonical amplitude, though in other cases the reverse was true (Tables 2 and 3 and Supplementary Section 10). Consequently, we recommend that researchers explore multiple ways of modeling the BOLD signal, particularly including gamma variate modeling in MDD, before concluding their experiment has low reliability. It may also be helpful for software for real-time analysis of fMRI data to implement alternative, potentially more reliable ways of characterizing BOLD responses in real-time.

Increasingly, the functional differentiation of sub-regions of subcortical structures such as the amygdala has been acknowledged as important for fMRI [49–52]. The comparison of test–retest reliability estimates obtained on the feedback signal averaged over the whole amygdala versus these same estimates computed voxelwise in the neurofeedback dataset suggest non-uniformity across the amygdala in signal reliability as well; the extent to which these differences explain previous results localizing function to subregions is unclear. Thus, we suggest it may be useful to use a voxel-wise or subregion approach to estimating test–retest reliability. Indeed, this method reveals significantly large clusters of voxels with excellent test–retest reliability in the left amygdala which could be used as masks for neurofeedback targets; our method is easily feasible for new studies. Excellent reliability, which is a prerequisite for clinical translation, was not attained in our dataset, using the more common computation of median ICCs for each ROI (e.g., as recommended by Caceres et al. (2009) [5]) (see Tables 1 and 4).

Reliability is generally considered a prerequisite for validity [56]. However, the choice of different modeling of the BOLD signal and selecting voxels of interest on a reliability threshold might have an impact on the effect size of the construct measured. We, therefore, recommend that researcher compare their effect size between their new more reliable method and their original method (see Supplementary Section 18 for an example).

Contrary to our hypotheses, we did not find that adding covariates to the model, including the scanner on which participants were run and severity, which did change as a function of intervention, improved test–retest reliability in these datasets (Supplementary Section 10) in ROI-based or whole-brain analyses (Supplementary Section 16). That said, covariates may still be useful to include in other datasets––we recommend exploring this option further before dismissing their utility.

Reliability did vary by whether the entire sample or only patient’s data were included and by whether or not participants were trained on the task, supporting the potential utility of quantifying reliability on tasks and populations that are relevant for the clinical application intended (Tables 2 and 3 and Supplementary Sections 11 and 17).

There are several limitations of this review and analyses. As we have focused only on MDD, it is unclear whether our conclusions apply transdiagnostically. Improving reliability may require different strategies in other diseases, such as Parkinson’s, due to age-related atrophy, increased movement, and differences in neurovascular coupling [53, 54]. There are many fMRI-based metrics we could have examined, including functional connectivity, volumetric measures, and resting-state designs, which all provoke unique considerations for optimizing test–retest reliability, some of which have been explored elsewhere [55]. We believe that functional connectivity results would still be dependent on task, regions of interest, and population [56]. Here, we focused on a regional BOLD activity as it is a common feature of prediction and neurofeedback studies. Our published data sets had relatively small number of subjects. This is typical for most clinical fMRI studies but does raise the concern that the sample is too small and underpowered. We recognize that until these results are replicated in an independent sample, they are specific to these two data sets. Our hope is that other teams can extend these results to other situations. Therefore, we strongly encourage the replication of these results.

## Conclusions

To summarize, demonstrating that mechanistic indices are reliable is important before their clinical adoption in prediction or treatment–development. The literature in these areas has implicitly accepted this assumption without testing it. Other non-clinical fMRI studies have shown many of the regions targeted in clinical fMRI studies have fairly low test–retest reliability, which was largely replicated using the most common analytic techniques in our datasets. Yet, we have suggested a few principles that appear to improve the test–retest reliability of the obtained mechanistic signals, have shown their feasibility in two previously published fMRI data sets, and have made code publicly available so that researchers with minimal mathematical and programming knowledge can implement them. Wider adoption of these methods could help to realize the potential of clinical fMRI and could extend to improving psychometrics for other time-varying mechanistic indices.

## Supplementary information

Supplement to Importance of test-retest reliability for promoting fMRI based screening and interventions in major depressive disorder
