# Correction: Importance of test–retest reliability for promoting fMRI based screening and interventions in major depressive disorder

**DOI:** 10.1038/s41398-021-01544-y

**Published:** 2021-08-17

**Authors:** Laurie Compère, Greg J. Siegle, Kymberly Young

**Affiliations:** Department of Psychiatry, University of Pittsburgh School of Medicine, Western Psychiatric Institute and Clinic, Pittsburgh, PA USA

**Keywords:** Depression, Neuroscience

Correction to: *Translational Psychiatry* 10.1038/s41398-021-01507-3, published online 10 July 2021

The original version of this article unfortunately contained mistakes in Tables 1 and 4. The correct tables are given below. The original article has been corrected.

**Table 1:** Table of mean, standard deviation and median values of ICCs for each sample, reactivity model, and ROI.

Population	Reactivity model	Amygdala	DLPFC	rACC	sgACC liberally thresholded	sgACC conservatively thresholded
Controls & patients	Canonical amplitude	0.11 (±0.09); 0.11	0.24 (±0.16); 0.26	0.09 (±0.10); 0.09	0.15 (±0.08); 0.13	0.17 (±0.09); 0.18
	Amplitude	0.23 (±0.14); 0.22	0.12 (±0.11); 0.12	0.11 (±0.10); 0.12	−0.01 (±0.13); −0.04	−0.04 (±0.14); −0.08
	Area under the curve	0.13 (±0.14); 0.12	0.08 (±0.10); 0.07	0.03 (±0.11); 0.03	−0.03 (±0.09); −0.04	−0.06 (±0.10); −0.07
	Onset delay	0 (±0.09); −0.01	0.01 (±0.09); 0	0 (±0.10); 0	0 (±0.08); 0.01	−0.01 (±0.10); 0
	Rise decay	0 (±0); 0	0 (±0); 0	0 (±0); 0	0 (±0); 0	0 (±0); 0
	Height	0.08 (±0.10); 0.09	0.21 (±0.15); 0.23	0.13 (±0.12); 0.14	0.16 (±0.12); 0.17	0.18 (±0.12); 0.23
Patients	Canonical amplitude	0.09(±0.11); 0.11	0.22 (±0.16); 0.23	0.08 (±0.14); 0.08	0.10 (±0.12); 0.07	0.14 (±0.15); 0.12
	Amplitude	0.22 (±0.15); 0.22	0.11 (±0.13); 0.11	0.10 (±0.13); 0.11	−0.06 (±0.15); −0.07	−0.08 (±0.14); −0.08
	Area under the curve	0.13 (±0.14); 0.12	0.6 (±0.12); 0.03	0.03 (±0.13); 0.04	−0.08 (±0.13); −0.08	−0.10 (±0.13); −0.09
	Onset delay	−0.01 (±0.12); −0.01	0.01 (±0.12); 0	−0.01 (±0.13); −0.01	0.02 (±0.11); 0.02	0.01 (±0.12); 0.05
	Rise decay	0 (±0); 0	0 (±0); 0	0 (±0); 0	0 (±0); 0	0 (±0); 0
	Height	0.09 (±0.12); 0.08	0.22 (±0.16); 0.23	0.12 (±0.15); 0.13	0.16 (±0.17); 0.18	0.17 (±0.17); 0.21

Mean (±standard deviation); median.

**Table 4:** Table of mean, standard deviation and median values of ICCs for each sample, preprocessing, and first level parameter with cluster correction applied.

Preprocessing		TBV style	Standard
Without training - Control - Baseline	Canonical amplitude	−0.07 (±0.21); −0.09	0.01 (±0.24); 0
	Amplitude	0.29 (±0.2); 0.3	0.26 (±0.22); 0.27
	Area under the curve	0.02 (±0.21); 0.01	0.21 (±0.23); 0.18
	Onset-delay	−0.03 (±0.23); −0.05	−0.11 (±0.20); −0.14
	Rise-decay	NA (±NA); NA	NA (±NA); NA
	Height	0.36 (±0.23); 0.33	0.17 (±0.38); 0.24
With training - Experimental - Transfer	Canonical amplitude	−0.11 (±0.21); −0.12	0.08 (±0.21); 0.09
	Amplitude	0.3 (±0.18); 0.31	0.26 (±0.21); 0.25
	Area under the curve	0.06 (±0.20); 0.07	0.13 (±0.18); 0.13
	Onset-delay	0.02 (±0.24); −0.02	−0.05 (±0.24); −0.13
	Rise-decay	NA (±NA); NA	NA (±NA); NA
	Height	0.52 (±0.19); 0.56	0.35 (±0.28); 0.34

Mean (±standard deviation); median.

