# Supplementary material for: Importance of test–retest reliability for promoting fMRI based screening and interventions in major depressive disorder
Source: Transl Psychiatry. 2021 Jul 10;11:387. doi: 10.1038/s41398-021-01507-3 (PMC8272717; doi:10.1038/s41398-021-01507-3)
Supplement: Supplementary file 1 — Supplement to Importance of test-retest reliability for promoting fMRI based screening and interventions in major depressive disorder [file 41398_2021_1507_MOESM1_ESM.pdf]

Supplement to Importance of test-retest reliability for promoting fMRI based screening and interventions in major depressive disorder

Laurie Compère, PhD, Greg J. Siegle, PhD, Kymberly Young, PhD

Contents

Supplement section 1: Computation of voxelwise ICCs using different tools 3

Supplement section 2: Studies Examining neuroimaging biomarkers of pharmacotherapy and psychotherapy outcomes in Major Depressive Disorder and mention of test-retest reliability of the studies 5

Supplement section 3: rt-fMRI-nf studies in Major Depressive Disorder and mention and possibility of test-retest reliability 16

Supplement section 4: The particular case of functional localizers 18

Supplement section 5: Details regarding datasets used 19

Supplement section 6: A Priori Region Definitions 20

Supplement section 7: Table of number of contiguous voxels for used cluster correction and p values associated for each reliability threshold, ROI, group for treatment outcome data set 22

Supplement section 8: Table of Kruskal Wallis tests' output for each sample, reactivity model with and without covariates, and ROI with Bonferroni correction applied. 25

Supplement section 9: Table of Kruskal Wallis tests' output for each sample, reactivity model with and without covariates, and ROI with Bonferroni correction applied, without rise decay. 26

Supplement section 10: Average group ranks and confidence intervals for semi partial correlations distributions for each model in each ROI in each data set. 27

Supplement section 11: Table of rescaled average group ranks of semi partial correlations for each sample, first level parameter, and ROI, with and without covariates (fMRI activation task dataset) 31

Supplement section 12: Methodological choice to fit gamma variates 32

Supplement section 13: Table of number of contiguous voxels for used cluster correction and p values associated for each reliability threshold, ROI, group and preprocessing for neurofeedback dataset 33

Supplement section 14: Table of Kruskal Wallis tests' output for each sample, reactivity model with and without covariates in the left amygdala with Bonferroni correction applied. 35

Supplement section 15: Table of Kruskal Wallis tests' output for each sample, reactivity model with and without covariates in the left amygdala with Bonferroni correction applied, without rise decay. 36

Supplement section 16: Voxelwise benefit from adding covariates (fMRI activation task dataset) 37

Supplement section 17: Table of rescaled average group ranks of semi partial correlations for each sample, preprocessing, first level parameter, and ROI, with and without covariates (rtfMRI-nf dataset).. 38

Supplement section 18: Tables of A. Reliability estimates in the patient group and the magnitude of change of activation before and after CBT (fMRI activation dataset) and B. their correlations, and C. Whole brain figures of the voxels with 1.reliability above 0.4, 2.magnitude of change before/after CBT above 0.24 with height parameter in patients and 3.mask of all the ROIs.. 39

SUPPLEMENTAL REFERENCES 43

Supplement section 1: Computation of voxelwise ICCs using different tools

AFNI's 3dLME and 3dICC functions (Chen et al., 2013) and the FMRELI Matlab toolbox, which is specifically designed for fMRI reliability analyses (Fröhner et al., 2019), support this metric. Both AFNI's 3dLME/3dICC functions and the FMRELI Matlab's toolbox allow computation of type 2 and 3 ICCs. Of these, we have only examined AFNI's 3dLME; it has moderate convergence, which differed by brain region, with well-validated computations implemented in via Matlab (see below). There are also different ways that co-variates can be handled within reliability models. Thus, we note that simply stating that ICC values were computed is unlikely to generalize across analyses – it is at least essential to cite the used software. For the current manuscript, given observed discrepancies, we stayed with a Matlab ICC implementation which we could verify, line for line, with textbook computations, and retreated from the ICC computation to more uniformly accepted methods for computations that necessitated the use of covariates.

Since AFNI's 3dLME function also allows to compute voxel wise ICCs by using a Bayesian approach (-ICCb) to be preferred to an older approach (-ICC), we used that function on the first level canonical amplitude as an example to be able to compare the results obtained from different tools (AFNI and our Matlab function). Using 3dLME, we just added subjects as a random factor, following the example in the online documentation to get an ICC close to type 3, the one that we computed with Matlab. Our Matlab function (icc.m, available from https://github.com/PICANlab/Reliability_toolbox), compute ICC(3,1) as described in Shrout and Fleiss (1979). Then, we computed correlations between ICCs values originated by AFNI and Matlab in all voxels or in ROI:

Sample	Correlations between ICCs values from Matlab	And from AFNI using Bayesian approach (-ICCb)	And from AFNI using old approach (-ICC)
Patients & controls	On all voxels	r=0.7	r=0.62
	Voxels within the amygdala	r=0.76	r=0.71
	Voxels within DLPFC	r=0.87	r=0.8
	Voxels with rACC	r=0.69	r=0.49
	Voxels within the sgACC (conservatively thresholded)	r=0.90	r=0.49
	Voxels within all ROIs	r=0.84	r=0.77
Patients	On all voxels	r=0.58	r=0.35
	Voxels within the amygdala	r=0.62	r=NA
	Voxels within DLPFC	r=0.76	r=0.49
	Voxels with rACC	r=0.31	r=0.13
	Voxels within the sgACC	r=0.66	r=0.27

	(conservatively thresholded)		
	Voxels within all ROIs	r=0.63	r=0.38

We wanted to share those results to show that using different tools to get ICC values is likely to give variable results, which also vary based on region.

Supplement section 2: Studies Examining neuroimaging biomarkers of pharmacotherapy and psychotherapy outcomes in Major Depressive Disorder and mention of test-retest reliability of the studies

Reference	Treatment(s)	Biomarker	Findings	Mention of signal reliability	Possibility to test signal reliability
Sheline et al.(2001)	Sertraline	Amygdala	Decreased activation following treatment	No	Yes
Davidson, et al. (2003)	Venlafaxine	ACC	Greater activation at baseline associated with better treatment response	No	Yes
Fu et al. (2004)	Fluoxetine	ACC, ventral striatum, cerebellum	Reduction of dynamic range associated with symptoms improvement	No	Yes
Canli et al. (2005)	None	Amygdala	Amygdala activation at baseline predicts symptom improvement	No	No
Schaefer, et al. (2006)	Venlafaxine	Prefrontal, temporal and parietal cortices, insula, basal ganglia and hippocampus	Normalized activation after treatment	No	Yes
Siegle, Carter, & Thase (2006)	CBT	sgACC and amygdala	Low and high, respectively, activation is associated with greater symptom improvement after therapy	No	No

Anand, et al. (2007)	Sertaline	Amygdala and ACC	Decrease activation in limbic regions and increased connectivity with the ACC after treatment	No	Yes
Chen et al. (2007)	Fluoxetine	ACC	Greater activation at baseline predict faster rates of symptom improvement	No	No
Fales et al. (2007)	Escitalopram	DLPFC	Enhanced activation following treatment	No	Yes
Fitzgerald et al. (2007)	TMS	Middle frontal gyrus, left precuneus, left precentral gyrus, left medial frontal gyrus, right inferior frontal gyrus	Decreased activation after low frequency treatment in middle frontal gyrus and left precuneus in respondents – Increased activation after high frequency treatment in left prefrontal gyrus, left medial frontal gyrus, right inferior frontal gyrus in respondents	No	Yes
Fu et al. (2007)	Fluoxetine	Hippocampus and extrastriate cortex	Greater activation following treatment and associated with	No	Yes

			symptom improvement		
Langenecker et al. (2007)	S-citalopram	Insula, right middle frontal gyrus, left inferior frontal gyrus, amygdala and cerebellar vermis	Greater activation at baseline associated with symptoms improvement	No	Yes
Robertson et al. (2007)	Bupropion	Amygdala	Reduced activation associated with symptom improvement	No	Yes
Walsh et al. (2007)	Fluoxetine	dACC, left middle frontal and lateral temporal cortices	Recuced activity at baseline associated with symptom improvement	Yes (discussion section)[a]	Yes
Fu et al. (2008)	CBT	dACC	Reduced activation at baseline associated with symptom improvement	No	Yes
Benedetti et al. (2009)	Venlafaxine	Right medial frontal gyrus	Decreased activation following treatment was associated with symptom improvement	No	Yes
Costafreda, et al. (2009)	CBT	ACC, superior and middle frontal cortices, paracentral cortex, superior	Activation contributed to prediction of remission	No	No

		parietal cortex, precuneus and cerebellum			
Dichter et al. (2010)	Behavioral Action Therapy	Paracingulate gyrus	Activation was prognostic for depressive symptom change after psychotherapy	No	Yes
Forbes et al., 2010	CBT and SSRI	Striatum and mPFC	Final levels of severity symptoms were related to pretreatment striatal reactivity and greater striatal and lower mPFC activity was prognostic for anxiety symptom reduction	No	No
Keedwell et al. (2010)	Various antidepressants	Right visual cortex and right sgACC	Greater baseline activity associated with clinical improvement after treatment	No	Yes
Lemogne et al. (2010)	Various antidepressants	Left DLPFC	Reduced activation following treatment	No	Yes
López-Solà et al. (2010)	Duolexetine	pACC, right prefrontal cortex, pons	Clinical improvement associated with reduced activation	No	Yes
Roy et al. (2010)	Citalopram	Ventromedial prefrontal	Greater activation at baseline	No	Yes

		cortex and ACC	associated with symptom improvement		
Victor, et al. (2010)	Sertaline	Amygdala	Decreased activation after treatment	No	Yes
Wagner et al. (2010)	Citalopram, reboxetine	Amygdala, hippocampus	Decreased activation after citalopram treatment	No	Yes
Frodl et al. (2011)	Mirtazapine, venlafaxine	Left fusiform gyrus, right rolandic operculum	Increased activation in the left fusiform gyrus at baseline was associated with a better response to venlafaxine and smaller activation in the right rolandic operculum was related to better response to mirtazapine	No	Yes
Light et al. (2011)	Venlefaxine, fluoxetine	Ventrolateral prefrontal cortex	Reduced activity at baseline is associated with anhedonia reduction	No	Yes
Ritchey, et al. (2011)	CBT	Ventromedial prefrontal cortex	Increased activity at baseline associated with symptom improvement	No	Yes
Samson et al. (2011)	Mirtazapine, venlafaxine	dmPFC, posterior cingulate cortex,	Greater activation at treatment associated	No	Yes

		superior frontal gyrus, caudate nucleus and insula	with better treatment response		
Arnone et al. (2012)	Citalopram	Amygdala	Reduced activation following treatment	No	Yes
Godlewska, et al. (2012)	Escitalopram	Amygdala	Reduced activity after treatment	No	No
Rosenblau et al. (2012)	Escitalopram	Amygdala, prefrontal cortex	Decreased activation following treatment	No	Yes
Ruhé, et al. (2012)	Paroxetine	Amygdala	Lower activation associated with better response to treatment after	No	Yes
Siegle et al. (2012)	CBT	sgACC	Reduced activation at baseline associated with greater symptom improvement	Yes[b]	Yes
Stoy et al. (2012)	Escitalopram	Ventral striatum	Increased activation following treatment	No	Yes
Tao et al. (2012)	Fluoxetine	Amygdala, orbitofrontal cortex and sgACC	Decreased activation after treatment	Yes (discussion section)[c]	Yes
Wang et al. (2012)	Fluoxetine	Insula, left ACC and middle frontal gyrus	Decreased activation in insula and left ACC and greater in the middle frontal gyrus following treatment	No	Yes

Furey et al. (2013)	Scopolamine	Middle occipital cortex	Increased activation at baseline was prognostic for symptoms improvement	No	Yes
Heller et al. (2013)	Fluoxetineor venlafaxine	Nucleus accumbens	Greater activation following treatment associated with more self-reported positive affect	No	Yes
Miller et al. (2013)	Escitalopram	Midbrain, DLPFC, paracingulate, ACC, thalamus and caudate nuclei	Reduced activation at baseline correlated with greater improvement following treatment	No	No
Rizvi et al. (2013)	Fluoxetine and olanzapine	Premotor cortex	Increased activation at baseline in respondents was prognostic for symptom improvement	Yes but not reported (method section)[d]	Yes
Victor, et al. (2013)	Sertraline	pgACC	Increased correlation at baseline correlated with greater clinical improvement after treatment	No	Yes
Toki et al. (2014)	Various antidepressants	Left hippocampus	Increased activation associated with greater response treatment	No	No
Yoshimura et al. (2014)	CBT	vACC	Improvements in depressive	No	Yes

			symptoms were negatively correlated with its activity		
Fu et al. (2015)	Duloxetine	Posterior cingulate cortex	Increased activation following treatment	Yes (limitation section)[e]	Yes
Furey et al. (2015)	Scopolamine	sgACC and middle occipital cortex	Increased and decreased activation, respectively, associated with treatment response	No	Yes
Straub et al., 2015	CBT	sgACC	Activation before treatment related to therapeutic success	No	Yes
Williams et al. (2015)	Escitalopram, sertraline, venlafaxine	Amygdala	Decreased activation at baseline was associated with treatment response	No	Yes
Cullen et al. (2016)	Various antidepressants	Rostral and sgACC, insula, middle frontal cortex, right hippocampus and left cerebellum	Decreased activation in postral and sgACC and increased in , insula, middle frontal cortex, right hippocampus and left cerebellum associated with symptom improvement	No	Yes

Delaveau et al. (2016)	Agomelatine	DLPFC and precuneus	Activation at baseline was related to treatment response	No	Yes
Doerig et al. (2016)	CBT	Amygdala	Activity in this region pre-intervention is negatively correlated with the outcome	No	No
Godlewska, et al. (2016)	Escitalopram	ACC, insula, amydgala and thalamus	Reduced activity after treatment associated with treatment response	No	Yes
Gyurak et al. (2016)	Escitalopram, sertraline and venlafaxine	DLPFC and inferior parietal cortex	Increased DLPFC activation at baseline associated with remission and increased inferior parietal activation associated with remission for SSRI and the opposite pattern for SNRI	No	Yes
Opmeer et al. (2016)	-	Rostral ACC	Increased activation at baseline was prognostic for remission	No	Yes
Szczepanik et al. (2016)	Scopolamine	Amygdala	Increased activity at baseline was associated with	Yes (limitation section)[f]	No

			symptoms improvement		
Fang et al., (2017)	Transcutaneous vagus nerve stimulation	Insula	Activation level at first stimulation session associated with clinical improvement	No	No
Sankar, et al. (2017)	Duloxetine	Left inferior frontal activity	Decreased activation following treatment	No	Yes
Spies et al. (2017)	Escitalopram	Precuneus and PCC	Deactivation before treatment was related to change in symptoms after 2 weeks of treatment	No	No
Godlewska et al. (2018)	Escitalopram	pgACC	Activity before treatment was able to predict response status (responder vs non-responder) at the level of individual participant	No	No
Rubin-Falcone et al. (2018)	CBT	sgACC, medial prefrontal cortex, lingual gyrus	Increased activation following treatment associated with better treatment outcome	No	Yes

ACC: Anterior Cingulate Cortex; CBT: Cognitive Behavioral Therapy; dACC: dorsal Anterior Cingulate Cortex; DLPFC: DorsoLateral Prefrontal Cortex; dmPFC: dorsomedial Prefrontal Cortex; mPFC : medial Prefrontal Cortex ; MDD: Major Depressive Disorder; PCC : Posterior Cingulate Cortex; pgACC: pregenual Anterior

Cingulate Cortex; sgACC: subgenual Anterior Cingulate Cortex ; SSRI : selective serotonine reuptake inhibitor

[a] "Test-retest effects were accounted for by the healthy control group, who underwent the same scans at the same time points"
[b]"sgACC z scoresn and reactivity had moderate test-retest reliability in controls undergoing testing approximately 16 weeks apart (N=27; r=0.39 [P=0.04])
[c] "repeat fMRI assessment of healthy comparison subjects, as well as repeat assessment of the depressed adolescents, thus providing assessment of expected test-retest reliability"
[d] "For analyses of change over time, a higher level fixed effects analysis was run for each subject, contrasting parameter estimates within subject for the response to slides at the two time points of interest."
[e]"perhaps in part reflecting the poor test-retest reliability of amygdala response to these emotional faces [54], while resting-state fMRI data show greater robustness and reproducibility [55]. Test-retest reliability of a neuroimaging measure becomes particularly important in the development of biomarkers for prognosis and diagnosis [44]."
[f]"some investigators have raised concerns regarding the reliability of the BOLD signal (Boubela et al., 2015). Nevertheless, studies have found that emotional stimuli evoke a consistent pattern of responsivity over repeated sessions (Johnstone et al., 2005)."

Supplement section 3: rt-fMRI-nf studies in Major Depressive Disorder and mention and possibility of test-retest reliability

Reference	Neurofeedback	ROI	Mention of the reliability of the signal	Possibility to test signal reliability	How could they look at reliability
Linden et al. (2012)	Upregulation	Functional localizer of brain areas involved in the generation of positive emotions (e.g., VLPLC, insula)	No	Yes	Same regions selected by the localizer on different sessions
Zotev, et al., (2014)	Upregulation	Left amygdala (anatomical)	No	No	-
Young et al. (2014) [a]	Upregulation	Left amygdala (anatomical)	No	Yes	Reliability of fMRI signal in ROI
Yuan et al. (2014)[a]	Upregulation	Left amygdala (anatomical)	No	No	-
Zotev et al. (2016)[a]	Upregulation	Left amygdala (anatomical)	No	No	-
Hamilton et al. (2016)	Downregulation	Functional localizer of the salience network	No	No	-
Young et al. (2017)[b]	Upregulation	Left amygdala (anatomical)	No	Yes	Reliability of fMRI signal in ROI
Young, Misaki, et al. (2017)[b]	Upregulation	Left amygdala (anatomical)	No	Yes	Reliability of fMRI signal in ROI
Young et al. (2018)[b]	Upregulation	Left amygdala (anatomical)	No	Yes	Reliability of fMRI signal in ROI
MacDuffie et al. (2018)	Upregulation and downregulation	Functional localizer of ACC	No	No	-

Mehler et al. (2018)	Upregulation	Functional localizer of brain areas involved in seeing positive versus neutral pictures (e.g., insula and striatum)	No	Yes	Same regions selected by the localizer on different sessions

ACC: Anterior Cingulate Cortex; VLPFC: Ventrolateral Prefrontal Cortex
References associated with the same letter refer to the same data set

Supplement section 4: The particular case of functional localizers

In many rtfMRI-nf designs, a functional localizer is used to identify the brain region(s) to train (e.g., Linden et al., 2012). Use of functional localizers allows for dynamic adjustment of target areas/networks according to individual patterns, and for evaluation of areas that are most activated by the task at the individual level, but does not guarantee the stability of the signal, nor does it allow a coherent classification among individuals of a group on the basis of the magnitude of activity. Examining the stability of regions activated during a functional localizer task takes into account the variability of interindividual functional specialization, and allows researchers to observe how stable the region/network trained in participants is from one training session to another. Theoretically, regions trained in one session should result in these same regions being more activated during the next session. If the overlap between the target areas from one session to another is weak, it is possible that the training has not been successful. Thus, overlap of functionally defined areas from one session to another is one indication of the reliability of the training procedure. While to our knowledge, no rtfMRI-nf study has reported on the stability of the localizer, this provides neurofeedback researchers an opportunity to examine signal reliability, and we encourage researchers using functional localizers to report the stability/overlap of active voxels selected on different training days at the individual level. In contrast, the field of EEG-neurofeedback, which has broken through to clinical audiences, may have been successful, in part, because it is built on reliability studies of the EEG parameters they subject to neurofeedback (Harmonya et al., 1993; McEvoy et al., 2000; Pollock et al., 1991; Salinsky et al., 1991).

Supplement section 5: Details regarding datasets used

Neuroimaging treatment outcome dataset

The sample consisted of 57 patients with major depressive disorder and 34 healthy control participants. Participants were assessed twice, approximately 12-16 weeks apart, during which patients received SSRI medication or CT. Two different scanners were used (both 3T Siemens Trio). The fMRI task was a personal relevance rating task in which participants were asked to indicate the extent to which positive, neutral or negative words were relevant to them or their lives. In this slow event related task, only responses to negative words were analyzed. The University of Pittsburgh Institutional Review Board (IRB) approved this study and all participants gave written consent to participate. The full sample is described in Strege et al. (2020) and represents an augmented sample based on the that described in Siegle et al. (2012a), which provides a complete description of the CT design, population, fMRI task, and processing. We worked with Strege et al. (2020) criteria with the exception of one participant that was excluded due to too few trials preserved in the regression after trial by trial motion censuring and that only participant that have been scanned twice were considered.

Neurofeedback dataset

36 patients (18-55 years), unmedicated, meeting the DSM-IV-TR criteria for MDD and experiencing a major depressive episode participated in a neurofeedback clinical trial during which participants were asked to retrieve positive memories while attempting to increase their hemodynamic activity in the assigned region (illustrated by a thermometer). In this double-blind, placebo-controlled, randomized clinical trial, participants were randomly assigned to receive two sessions of neurofeedback training approximately one week apart, either from the left amygdala or from a parietal control region. The neurofeedback protocol consisted of six runs and no feedback was provided during the first and last runs (baseline and transfer runs, respectively). During each run, participants alternately performed 40-second blocks of rest, happy memories (upregulate condition), and count (backward from 300 by a given number). Feedback was provided only during happy conditions. Imaging was performed using a GE-Discovery MR750 3-T scanner equipped with a custom rtfMRI neurofeedback system. The Western Institutional Review Board approved this research protocol that was registered on ClinicalTrials.gov and participants gave consent to participate in the study. Please refer to Young, Siegle, et al. (2017) for a complete description of the design, population and fMRI task.

Supplement section 6: A Priori Region Definitions

For a priori regionwise analyses (described in Table S3 below and Figure 2 in the main manuscript) we used the following ROIs:

Three a priori regions for this study (amygdala, DLPFC, rACC) were defined anatomically using anatomical atlases rendered on the Colin-27 Montreal Neurological Institute canonical brain. The fourth (sgACC) was defined meta-analytically, as described below.

Amygdala

The full amygdala region, as defined in the AAL atlas was used, as shown below.

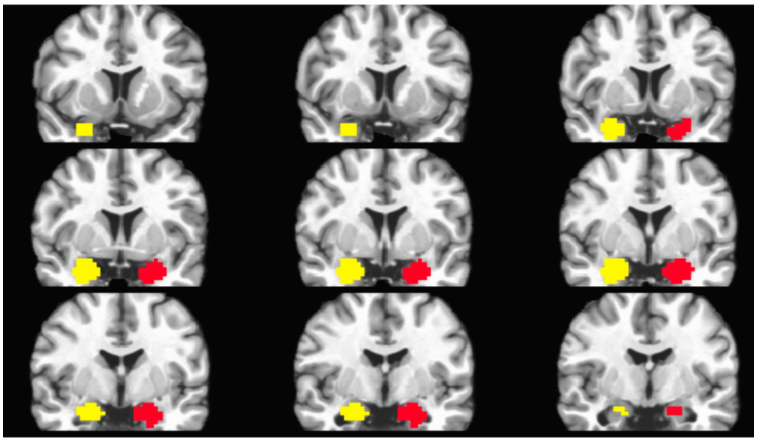

DLPFC

The DLPFC was defined as the middle frontal gyrus from AFNI's Talairach (TTatlas) map within 5<Z<37 to yield approximately the lateral BA9/46 region. Depressed and control participants differ on digit sorting as well as the personal relevance task used in this study in this region (Siegle et al., 2007).

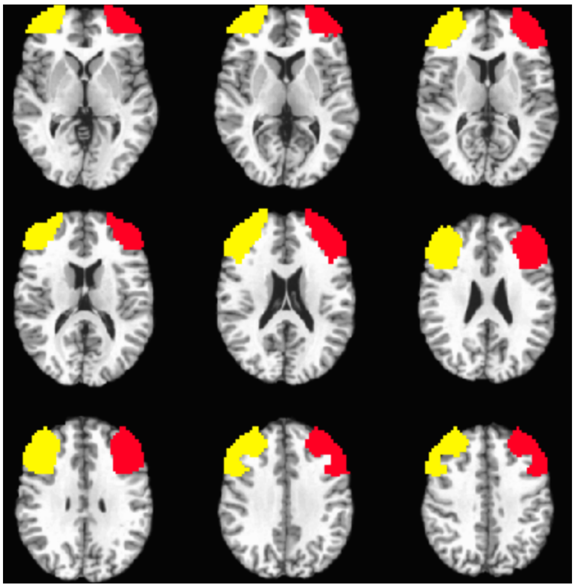

rACC

We defined an area of the rostral and subgenual cingulate including BA24 using Afni's Talairach atlas regions for the Anterior Cingulate and Cingulate Gyrus within

the range of Y>0 and Z<-15. The rationale for selection of this anatomically defined region is that BA24 in the rostral cingulate consistently predicts response in medication studies using PET (Brannan et al., 2000; Brody et al., 2001; Mayberg et al., 1997), fMRI (Chen et al., 2007; Davidson et al., 2003; Keedwell et al., 2010; Langenecker et al., 2007; Nitschke et al., 2009; Roy et al., 2010), and EEG (Pizzagalli et al., 2001). That said, as the predictive areas have been derived empirically in all of these studies, and do not always overlap, we chose a broader anatomically defined region which does encompass the majority of regions observed in previous studies.

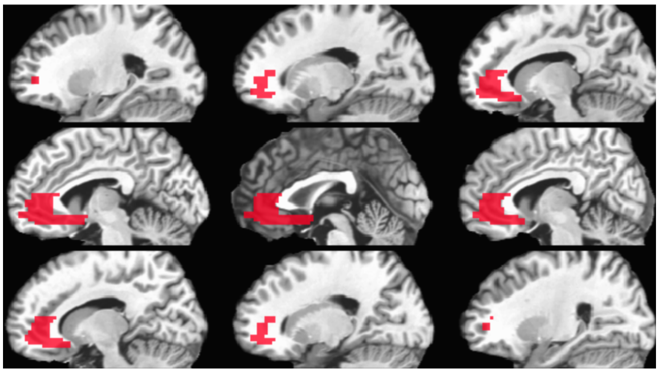

sgACC

Our previous studies of Cognitive Behavior Therapy (CBT) have specifically implicated a region of the subgenual cingulate in response to CBT (Siegle et al., 2012). We have recently submitted a meta-analysis of regions which predict response to both CBT and medication in which a region that almost entirely overlaps from that region can also be used to predict response to medication (Strege et al, submitted). We thus used that region. As is always the case, there is some level of arbitrariness to thresholding of meta-analytically defined maps. Our first instinct was to use a conservative thresholding (18 voxels) shown below. That said, we also considered a slightly more liberally thresholded version (33 voxels) as a way of examining the extent to which psychometric characteristics such as reliability could be used to help in defining anatomical masks.

Conservatively thresholded (18 voxels)

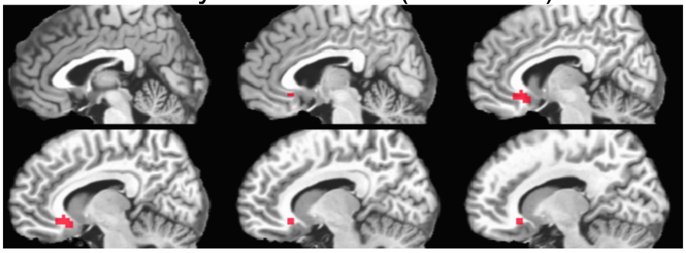

Liberally thresholded (33 voxels)

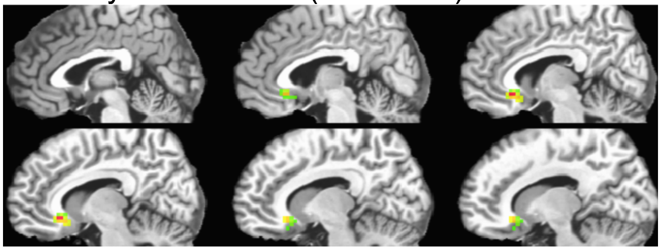

Supplement section 7: Table of number of contiguous voxels for used cluster correction and p values associated for each reliability threshold, ROI, group for treatment outcome data set

Population	First level model	Reliability level	Amygdala	DLPFC	rACC	sgACC liberally thresholded	sgACC conservatively thresholded
Controls & patients (N=91)	Canonical amplitude	**0.4**	1	1.8	1.1	-	-
		**0.6**	1	1	1	-	-
		**0.7**	1	1	1	-	-
		**0.75**	1	1	1	-	-
	Amplitude	**0.4**	2	1.6	1.1	-	-
		**0.6**	1	1	1	-	-
		**0.7**	1	1	1	-	-
		**0.75**	1	1	1	-	-
	Area under the curve	**0.4**	1	1.7	1.1	-	-
		**0.6**	1	1	1	-	-
		**0.7**	1	1	1	-	-
		**0.75**	1	1	1	-	-
	Onset delay	**0.4**	1	1.3	1.1	-	-
		**0.6**	1	1	1	-	-
		**0.7**	1	1	1	-	-
		**0.75**	1	1	1	-	-
	Rise decay	**0.4**	-	-	-	-	-
		**0.6**	-	-	-	-	-
		**0.7**	-	-	-	-	-
		**0.75**	-	-	-	-	-
	Height	**0.4**	1	1.8	1.1	-	-
		**0.6**	1	1	1	-	-
		**0.7**	1	1	1	-	-
		**0.75**	1	1	1	-	-
Patients	Canonical amplitude	**0.4**	2.6	6.7	4	-	-
		**0.6**	1	1	1	-	-

	**0.7**	1	1	1	-	-
	**0.75**	1	1	1	-	-
**Amplitude**	**0.4**	2	7.2	3.7	-	-
	**0.6**	1	1	1	-	-
	**0.7**	1	1	1	-	-
	**0.75**	1	1	1	-	-
**Area under the curve**	**0.4**	2.2	9.7	3.9	-	-
	**0.6**	1	1	1	-	-
	**0.7**	1	1	1	-	-
	**0.75**	1	1	1	-	-
**Onset delay**	**0.4**	1.6	2.6	2	-	-
	**0.6**	1	1	1	-	-
	**0.7**	1	1	1	-	-
	**0.75**	1	1	1	-	-
**Rise decay**	**0.4**	-	-	-	-	-
	**0.6**	-	-	-	-	-
	**0.7**	-	-	-	-	-
	**0.75**	-	-	-	-	-
**Height**	**0.4**	2.0	7.5	3.9	-	-
	**0.6**	1	1	1	-	-
	**0.7**	1	1	1	-	-
	**0.75**	1	1	1	-	-

Notes: After getting different numbers of voxels to use for cluster correction while running 3dClustsim two times in a row with a standard number of 2,000 simulations, we increased the number of iterations to 10,000 to obtain more stability in our results.
For 91 participants in whole sample group r=.4 yields p=.100025.
For 91 participants in whole sample group r=.6 yields p=.008479.
For 91 participants in whole sample group r=.7 yields p=.001219.
For 91 participants in whole sample group r=.75 yields p=.000338.
For 57 patients in patients group r=.4 yields p=.12475.
For 57 patients in patients group r=.6 yields p=.014007.
For 57 patients in patients group r=.7 yields p=.002535.
For 57 patients in patients group r=.75 yields p=.00082.

AFNI function 3dClustSim was not able to compute the number of voxels necessary for cluster correction with the masks of sgACC liberally and conservatively thresholded because of their small size (respectively, 33 and 18 voxels) so in this case, no correction was applied and on the first parameter rise decay rate in every ROI because data were badly fitted so there was no variance in the ICC values.
AFNI 3dFWHMx function for the residual ICC map with the first level parameter amplitude in the whole sample had a calculation error using ACF method with the original amygdala ROI so we dilated this ROI of one voxel in this only particular case.

Supplement section 8: Table of Kruskal Wallis tests' output for each sample, reactivity model with and without covariates, and ROI with Bonferroni correction applied.

**Population**	**Amygdala**	**DLPFC**	**rACC**	**sgACC liberally thresholded**	**sgACC conservatively thresholded**
**Controls & patients**	**H(11)=1414.67, p<0.001**	**H(11)=12717.07, p<0.001**	**H(11)=4794.14, p<0.001**	**H(11)=206.47, p<0.001**	**H(11)=118.32, p<0.001**
**Patients**	**H(11)=1233.13, p<0.001**	**H(11)=10371.75, p<0.001**	**H(11)=4477.55, p<0.001**	**H(11)=240.89, p<0.001**	**H(11)=136.93, p<0.001**

Note: Applying Bonferroni correction for 6 reactivity models with and without covariates (p<0.05/12=0.004).

Supplement section 9: Table of Kruskal Wallis tests' output for each sample, reactivity model with and without covariates, and ROI with Bonferroni correction applied, without rise decay.

Population	Amygdala	DLPFC	rACC	sgACC liberally thresholded	sgACC conservatively thresholded
**Controls & patients**	**H(9)=285.88, p<0.001**	**H(9)=4876.99, p<0.001**	**H(9)=644.19, p<0.001**	**H(9)=58.90, p<0.001**	**H(9)=40.55, p<0.001**
**Patients**	**H(9)=25.68, p=0.002**	**H(9)=1588.15, p<0.001**	**H(9)=190.42, p<0.001**	**H(9)=108.21, p<0.001**	**H(9)=67.20, p<0.001**

Note: Applying Bonferroni correction for 6 reactivity models with and without covariates ($p<0.05/10=0.005$).

Supplement section 10: Average group ranks and confidence intervals for semi partial correlations distributions for each model in each ROI in each data set.

<table>
<tr><th colspan="3">Siegle et al. (2012) data set</th></tr>
<tr><th>ROI</th><th>Controls & patients</th><th>Patients</th></tr>
<tr><td>Amygdala</td><td>

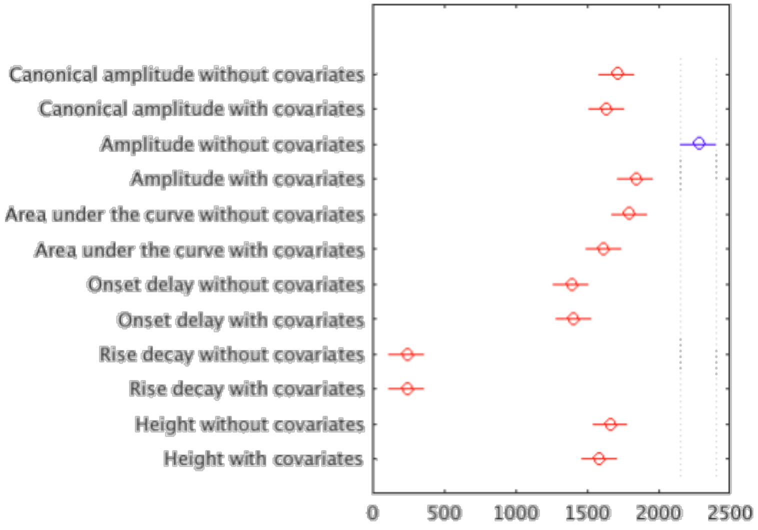

</td><td>

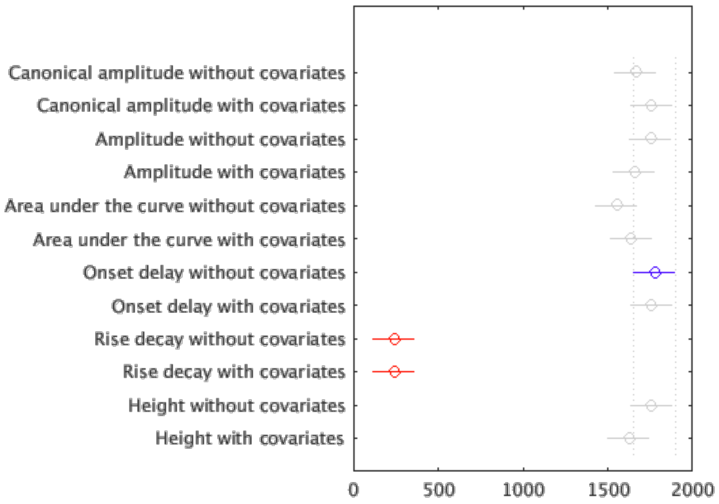

</td></tr>
</table>

DLPFC

rACC

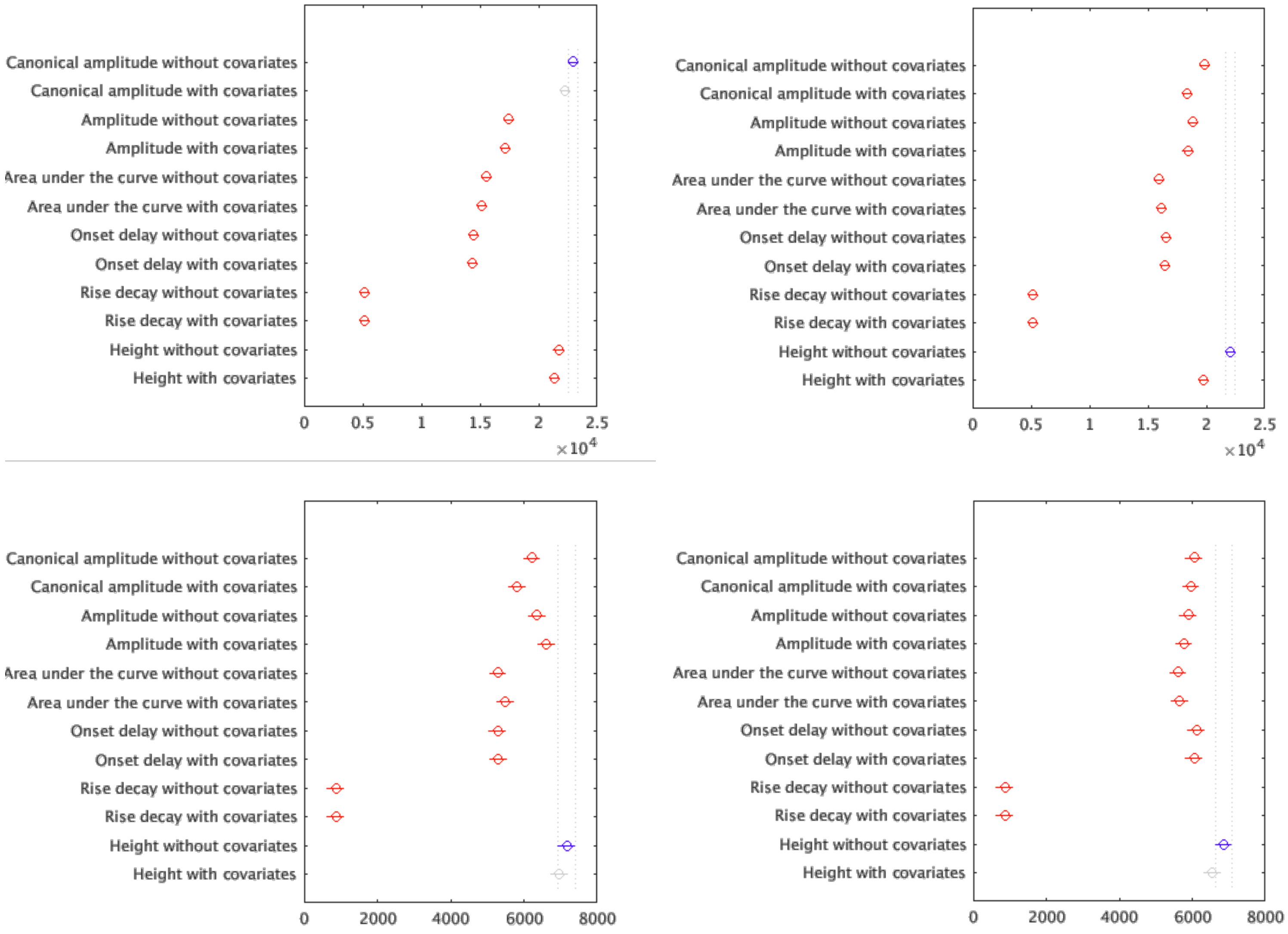
Canonical amplitude without covariates
Canonical amplitude with covariates
Amplitude without covariates
Amplitude with covariates
Area under the curve without covariates
Area under the curve with covariates
Onset delay without covariates
Onset delay with covariates
Rise decay without covariates
Rise decay with covariates
Height without covariates
Height with covariates
0 0.5 1 1.5 2 2.5
$\times 10^4$
0 2000 4000 6000 8000

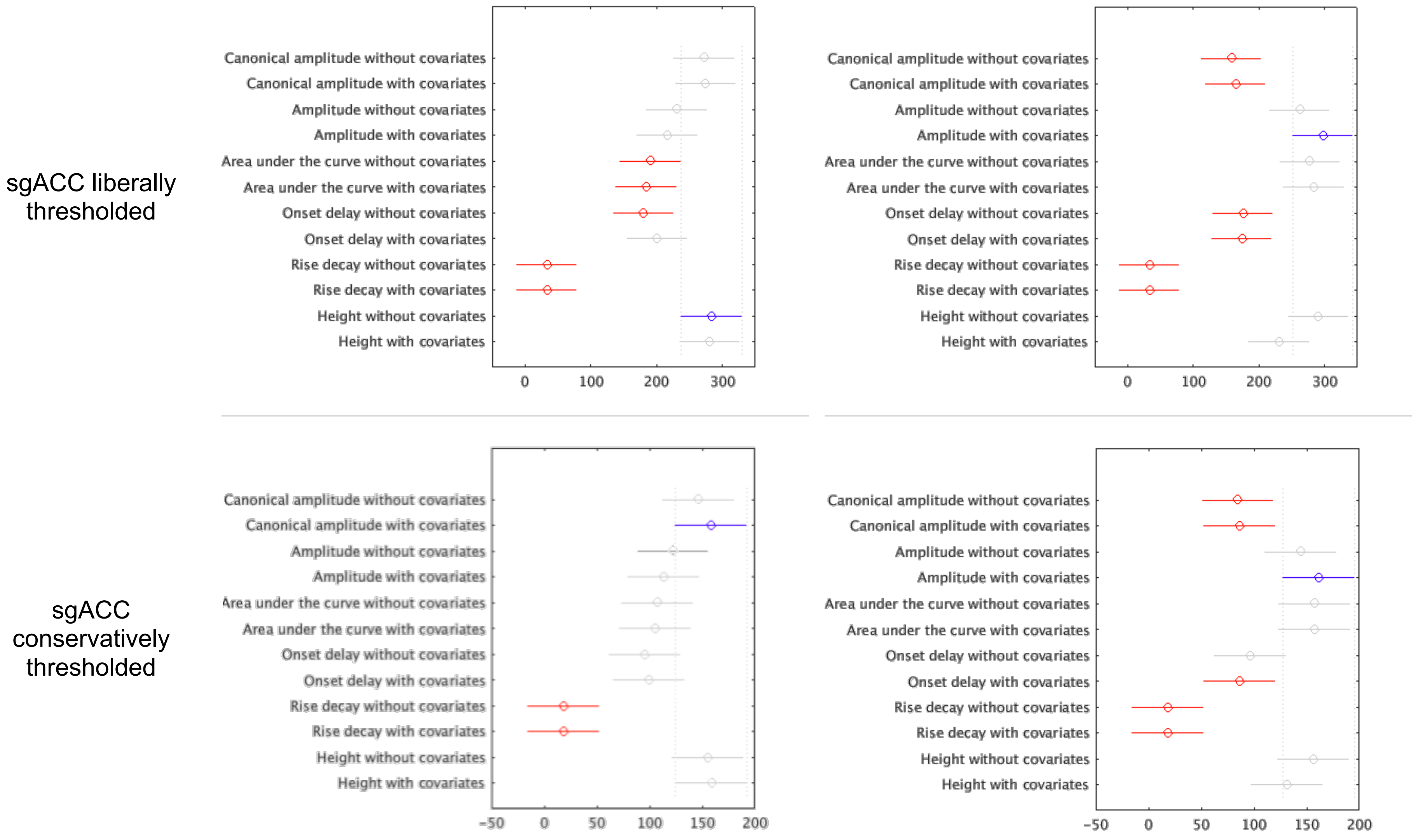

sgACC liberally thresholded
sgACC conservatively thresholded
Canonical amplitude without covariates
Canonical amplitude with covariates
Amplitude without covariates
Amplitude with covariates
Area under the curve without covariates
Area under the curve with covariates
Onset delay without covariates
Onset delay with covariates
Rise decay without covariates
Rise decay with covariates
Height without covariates
Height with covariates
0
100
200
300
-50
0
50
100
150
200

Young et al. (2017b) data set		
ROI	Signal without training	Trained signal
Amygdala	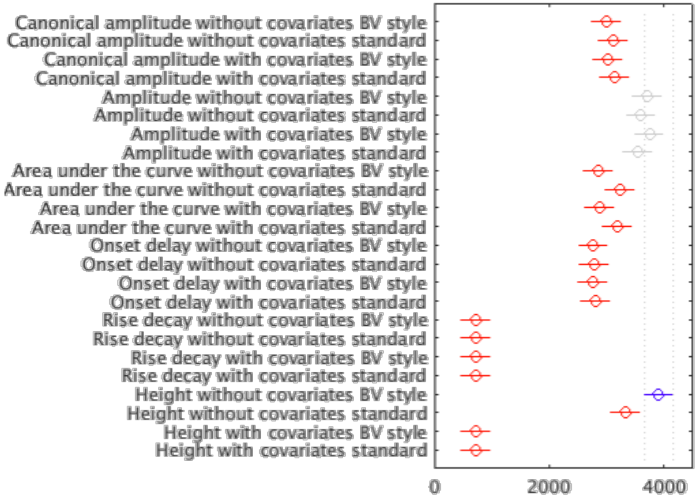	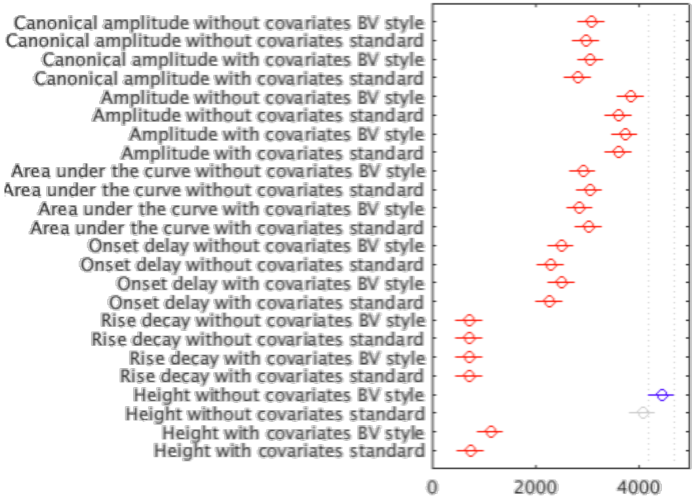

Supplement section 11: Table of rescaled average group ranks of semi partial correlations for each sample, first level parameter, and ROI, with and without covariates (fMRI activation task dataset)

Population	First level model	Covariates	Amygdala	DLPFC	rACC	sgACC liberally thresholded	sgACC conservatively thresholded
Controls & patients	Canonical amplitude	Without	7.07	8.6	7.2	8.16	8.25
		With	6.77	8.31	6.73	8.82	8.32
	Amplitude	Without	9.44	6.51	7.36	6.81	6.99
		With	7.62	6.42	7.65	6.33	6.56
	Area under the curve	Without	7.44	5.81	6.12	5.95	5.79
		With	6.68	5.68	6.36	5.87	5.6
	Onset delay	Without	5.75	5.39	6.1	5.28	5.45
		With	5.82	5.37	6.13	5.53	6.09
	Rise decay	Without	1	1.91	1	1.03	1.02
		With	1	1.91	1	1.03	1.02
	Height	Without	6.88	8.11	8.29	8.64	8.58
		With	6.56	7.97	8.06	8.88	8.51
Patients	Canonical amplitude	Without	6.89	7.41	7	4.72	4.79
		With	7.27	6.87	6.89	4.83	4.99
	Amplitude	Without	7.24	7.05	6.81	8.02	7.94
		With	6.85	6.88	6.68	8.98	9.01
	Area under the curve	Without	6.42	5.97	6.49	8.74	8.41
		With	6.77	6.05	6.54	8.74	8.59
	Onset delay	Without	7.34	6.18	7.07	5.38	5.32
		With	7.26	6.14	7	4.83	5.29
	Rise decay	Without	1	1.91	1	1.03	1.02
		With	1	1.91	1	1.03	1.02
	Height	Without	7.26	8.24	7.94	8.71	8.8
		With	6.72	7.39	7.58	7.33	7

Supplement section 12: Methodological choice to fit gamma variates

At the time of the analysis of the feedback signal, two methodological options were possible:

- Either averaging the signal over the blocks of interest and then computing the gamma variates parameters on the mean signal course (choice selected in the main manuscript) [1], or;
- Computing the gamma variates parameters over the signal course in each block and then average the parameters obtained for a run [2].

To make this decision, we tested both option on the left amygdala real-time feedback signal that we extracted from the output of previously used script for real-time preprocessing (Young et al., 2017b), see table below.

Sample	First level gamma variates parameter	Signal averaged over the blocks [1]	Parameter averaged over the run [2]
Signal without training – Baseline in control group	Onset delay	ICC = 0.54	ICC = 0.03
	Rise decay	ICC = -0.03	ICC = -0.07
	Height	ICC = 0.47	ICC = -0.03
Signal with training – Transfer in experimental group	Onset delay	ICC = -0.12	ICC = -0.13
	Rise decay	ICC = -0.06	ICC = -0.01
	Height	ICC = -0.12	ICC = 0.03

In conclusion, the second option resulted in poorer test retest reliability results, without this being explained by a poor signal fit. We therefore preferred the first methodology in the main version of the manuscript.

Supplement section 13: Table of number of contiguous voxels for used cluster correction and p values associated for each reliability threshold, ROI, group and preprocessing for neurofeedback dataset

Preprocessing			BV style	Standard
Without training Control - Baseline	Canonical amplitude	0.4	30.3	31.3
		0.6	4.1	4.9
		0.7	2.1	2.2
		0.75	1.4	1.5
	Amplitude	0.4	30.6	30.5
		0.6	4.3	4.4
		0.7	2.1	2.1
		0.75	1.4	1.4
	Area under the curve	0.4	35.6	36
		0.6	6.6	6.2
		0.7	2.8	2.5
		0.75	1.7	1.5
	Onset delay	0.4	27.8	28.7
		0.6	3.1	3.3
		0.7	1.7	1.7
		0.75	1.3	1.3
	Rise decay	0.4	-	-
		0.6	-	-
		0.7	-	-
		0.75	-	-
	Height	0.4	28.2	28.3
		0.6	3.3	3.4
		0.7	1.7	1.8
		0.75	1.3	1.3
With training Experimental Transfer	Canonical amplitude	0.4	28.3	29.4
		0.6	4.1	4.7
		0.7	1.7	1.8
		0.75	1.1	1.1
	Amplitude	0.4	26.3	26.2
		0.6	3.6	4.3
		0.7	1.7	1.8
		0.75	1.1	1.1
	Area under the curve	0.4	24.7	30
		0.6	3.9	5.2
		0.7	1.8	1.9
		0.75	1.1	1.1
	Onset delay	0.4	20	19.6
		0.6	2.4	2.4
		0.7	1.4	1.4
		0.75	1.1	1.1
	Rise decay	0.4	-	-
		0.6	-	-

	**0.7**	-	-
	**0.75**	-	-
**Height**	**0.4**	21.1	20.5
	**0.6**	2.8	2.7
	**0.7**	1.5	1.4
	**0.75**	1.1	1.1

Notes: After getting different numbers of voxels to use for cluster correction while running 3dClustsim two times in a row with a standard number of 2,000 simulations, we increased the number of iterations to 10,000 to obtain more stability in our results.
For 18 patients in experimental group r=.4 yields p=.100025.
For 18 patients in experimental group r=.6 yields p=.008479.
For 18 patients in experimental group r=.7 yields p=.001219.
For 18 patients in experimental group r=.75 yields p=.000338.
For 16 patients in control group r=.4 yields p=.12475.
For 16 patients in control group r=.6 yields p=.014007.
For 16 patients in control group r=.7 yields p=.002535.
For 16 patients in control group r=.75 yields p=.00082.
AFNI function 3dClustSim was not able to compute the number of voxels necessary for cluster correction on the first parameter rise decay rate because data were badly fitted so there was no variance in the ICC values.

Supplement section 14: Table of Kruskal Wallis tests' output for each sample, reactivity model with and without covariates in the left amygdala with Bonferroni correction applied.

Population	Amygdala
**Without training - Control - Baseline**	**H(23)=2964.56, p<0.001**
**With training - Experimental - Transfer**	**H(23)=3142.17, p<0.001**

Note: Applying Bonferroni correction for 6 reactivity models with and without covariates (p<0.05/12=0.004).

Supplement section 15: Table of Kruskal Wallis tests' output for each sample, reactivity model with and without covariates in the left amygdala with Bonferroni correction applied, without rise decay.

Population	Amygdala
**Without training - Control - Baseline**	**H(19)=1397.84, p<0.001**
**With training - Experimental - Transfer**	**H(19)=1702.57, p<0.001**

Note: Applying Bonferroni correction for 6 reactivity models with and without covariates (p<0.05/10=0.005).

Supplement section 16: Voxelwise benefit from adding covariates (fMRI activation task dataset)

Positive semi partial correlation difference computed with covariates versus without covariate in each voxel with the height gamma variates parameter A. For controls and patients. B. For patients only.

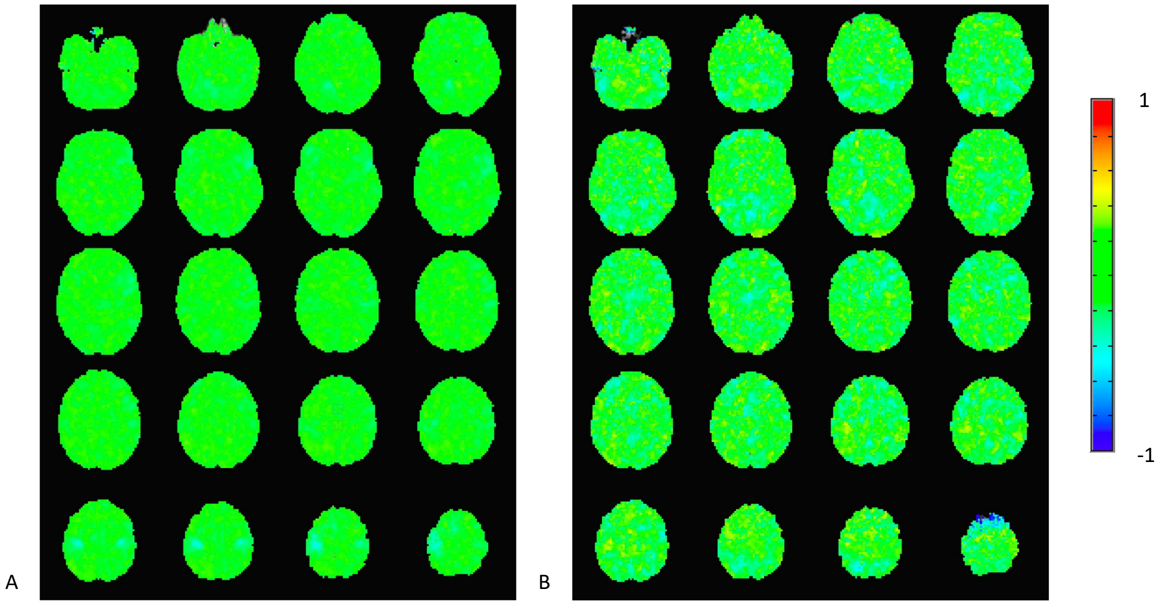

Supplement section 17: Table of rescaled average group ranks of semi partial correlations for each sample, preprocessing, first level parameter, and ROI, with and without covariates (rtfMRI-nf dataset)

Preprocessing			BV style	Standard
Without training Control - Baseline	Canonical amplitude	Without	7.01	7.29
		With	7.05	7.34
	Amplitude	Without	8.66	8.41
		With	8.75	8.26
	Area under the curve	Without	6.65	7.56
		With	6.71	7.42
	Onset delay	Without	6.47	6.49
		With	6.46	6.56
	Rise decay	Without	1.67	1.67
		With	1.67	1.67
	Height	Without	9.13	7.77
		With	1.67	1.67
With training Experimental Transfer	Canonical amplitude	Without	7.24	6.99
		With	7.19	6.62
	Amplitude	Without	9	8.46
		With	8.73	6.46
	Area under the curve	Without	6.83	7.14
		With	6.7	7.1
	Onset delay	Without	5.85	5.38
		With	5.89	5.32
	Rise decay	Without	1.71	1.71
		With	1.71	1.71
	Height	Without	10.39	9.53
		With	2.65	1.75

Supplement section 18: Tables of A. Reliability estimates in the patient group and the magnitude of change of activation before and after CBT (fMRI activation dataset) and B. their correlations, and C. Whole brain figures of the voxels with 1. reliability above 0.4, 2. magnitude of change before/after CBT above 0.24 with height parameter in patients and 3. mask of all the ROIs

	Amydgala (242 voxels)	DLPFC (2675 voxels)	rACC (865 voxels)	sgACC liberally thresholded (33 voxels)	sgACC conservatively thresholded (18 voxels)
**Mean ICC for the height reactivity model**	0.09	0.22	0.12	0.16	0.17
**Mean magnitude (cohen d) of change before-after CBT in the whole ROI**	-0.03	-0.13	-0.14	-0.02	-0.06
**% of voxels above an ICC threshold of 0.4**	0	14	1	6	6
**Mean magnitude (cohen d) of change before after CBT in the voxels above the ICC threshold of 0.4**	NA	-0.19	-0.17	0.25	0.20

A

Spearman correlations over ROIs	Mean ICC	% of voxels above threshold
**Mean magnitude**	-0.2	-0.1
**Mean magnitude in voxels above threshold**	-0.4	-0.32

B

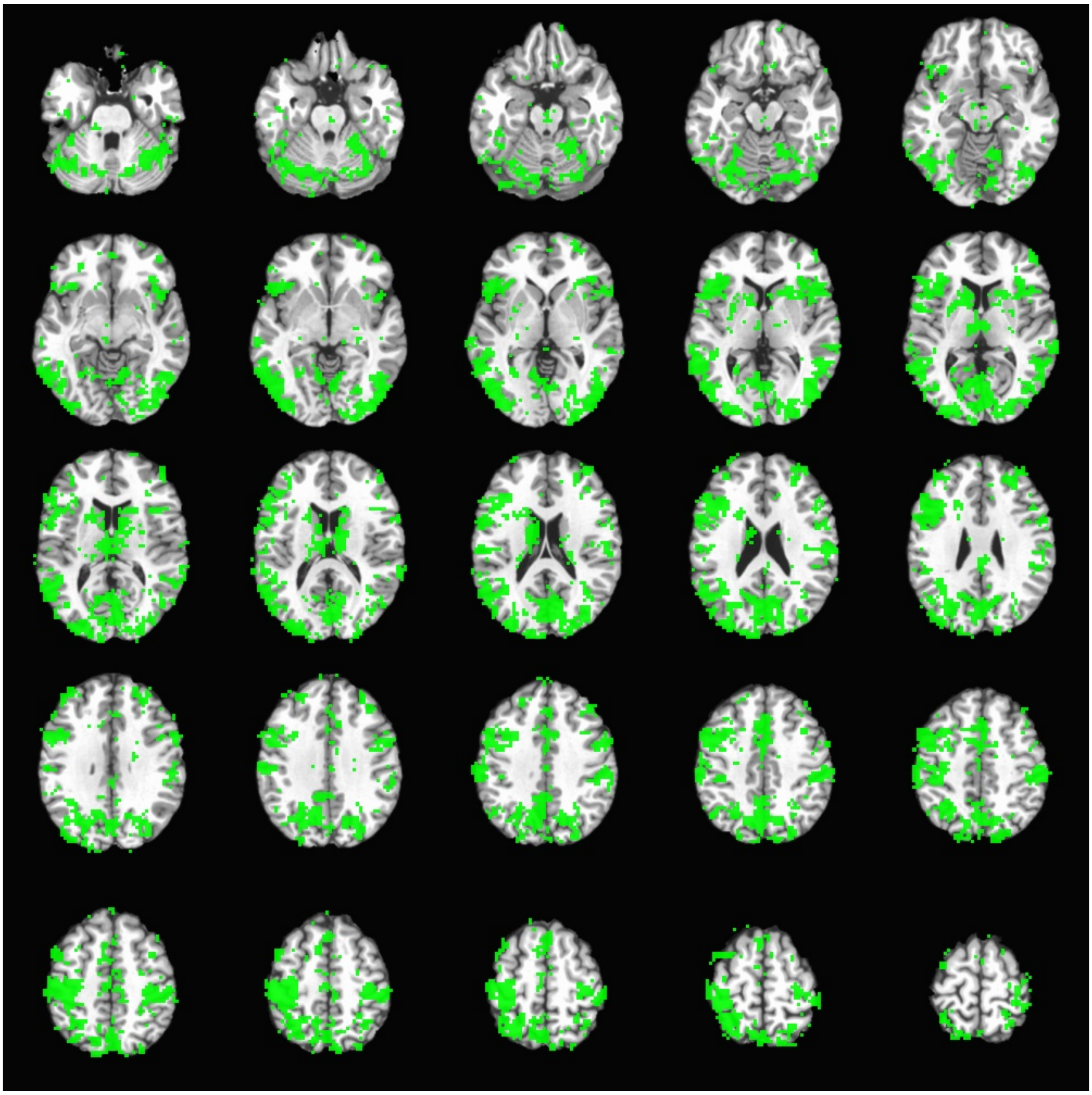

C.1. Whole brain figure of the voxels with reliability above 0.4 with height parameter in patients

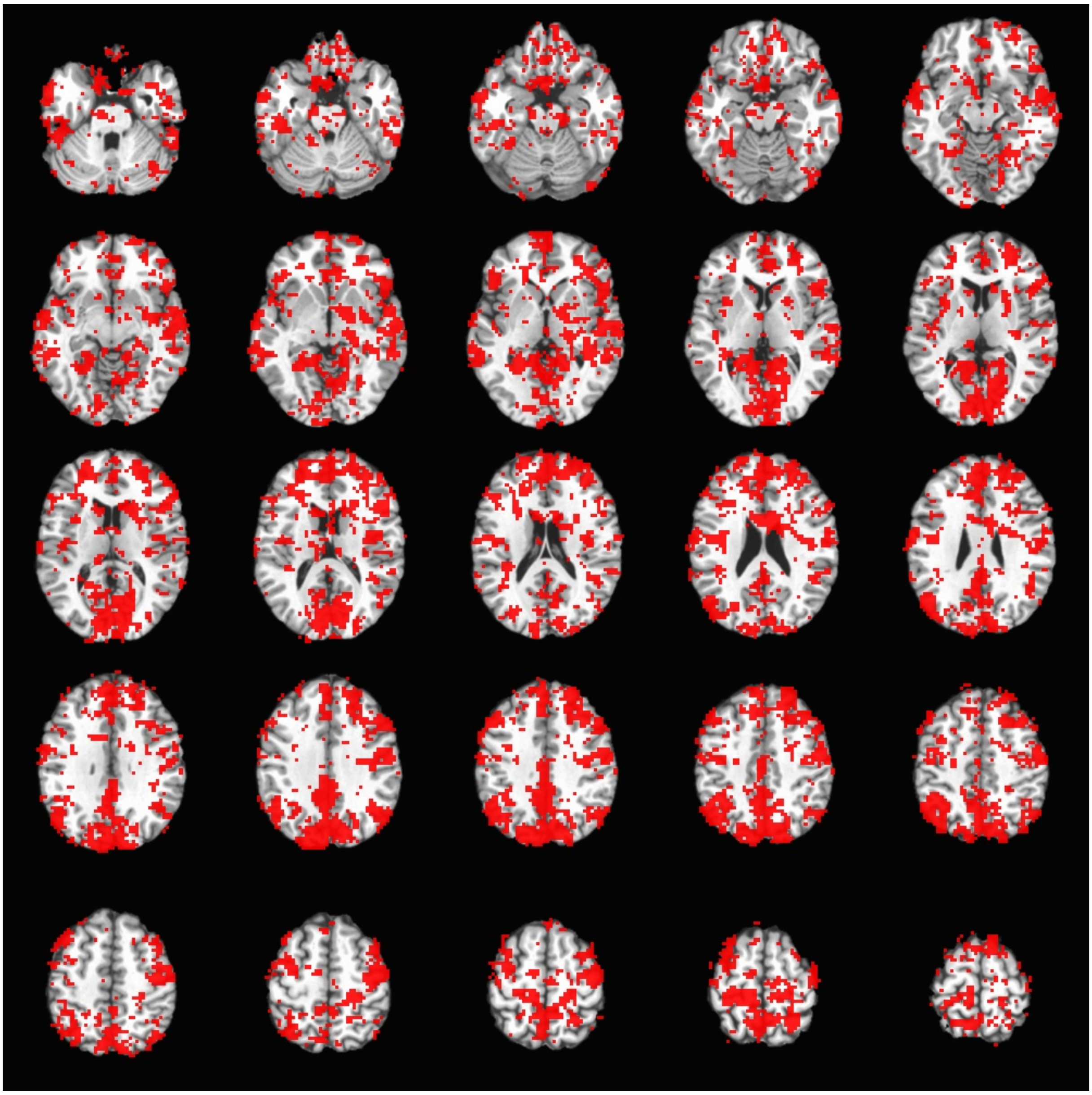

C.2. Whole brain figure of the voxels with magnitude of change before/after CBT above 0.24 with height parameter in patients

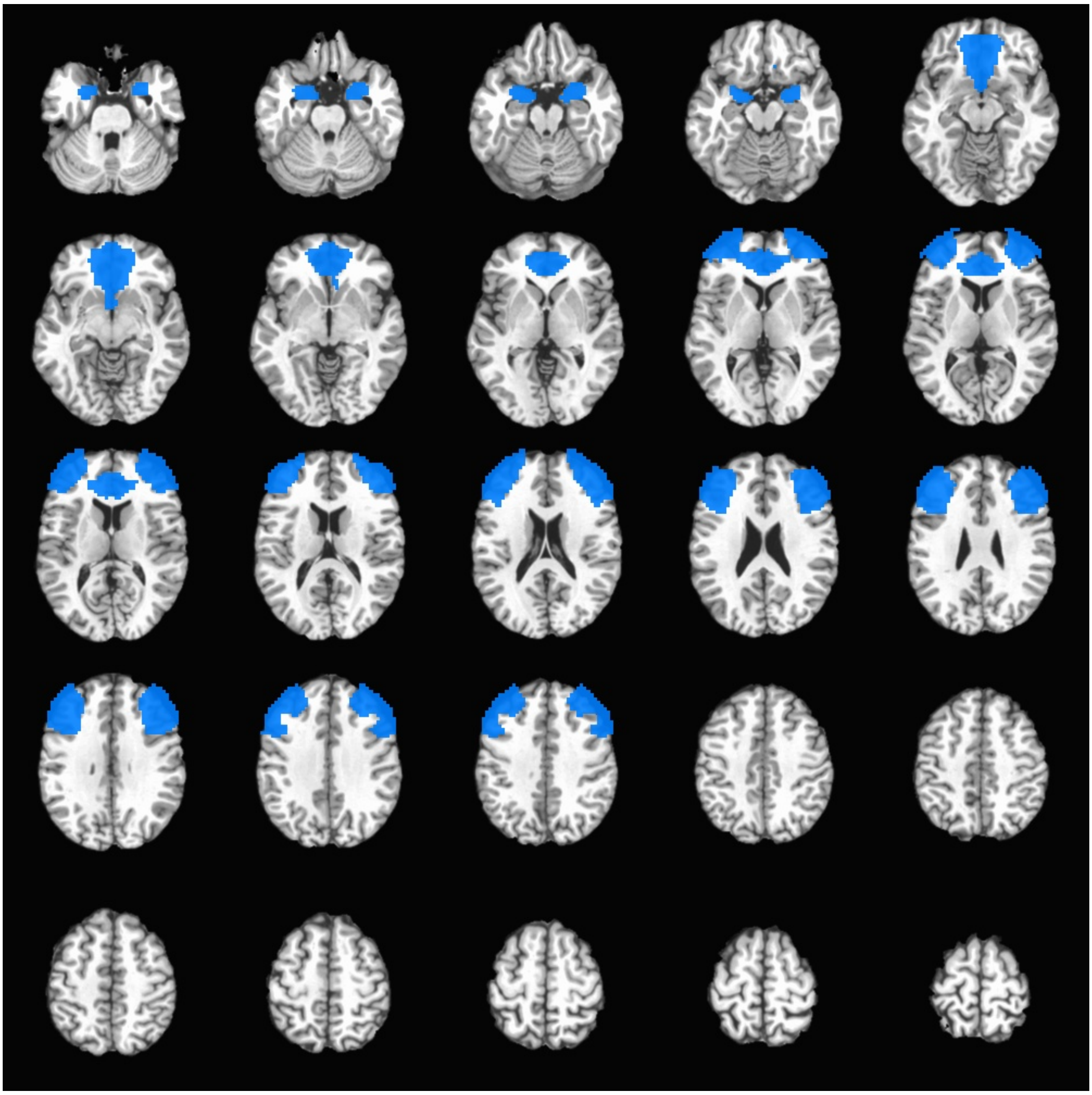

C.3. Whole brain figure of the mask of all the ROIs

SUPPLEMENTAL REFERENCES

Anand, A., Li, Y., Wang, Y., Gardner, K., Lowe, M.J., 2007. Reciprocal Effects of Antidepressant Treatment on Activity and Connectivity of the Mood Regulating Circuit: An fMRI Study. J. Neuropsychiatry Clin. Neurosci. https://doi.org/10.1176/jnp.2007.19.3.274

Arnone, D., McKie, S., Elliott, R., Thomas, E.J., Downey, D., Juhasz, G., Williams, S.R., Deakin, J.F.W., Anderson, I.M., 2012. Increased amygdala responses to sad but not fearful faces in major depression: Relation to mood state and pharmacological treatment. Am. J. Psychiatry. https://doi.org/10.1176/appi.ajp.2012.11121774

Benedetti, F., Radaelli, D., Bernasconi, A., Dallaspezia, S., Colombo, C., Smeraldi, E., 2009. Changes in medial prefrontal cortex neural responses parallel successful antidepressant combination of venlafaxine and light therapy. Arch. Ital. Biol.

Brannan, S.K., Mayberg, H.S., McGinnis, S., Silva, J.A., Tekell, J., Mahurin, R.K., Jerabek, P.A., Fox, P.T., 2000. 355. Cingulate metabolism predicts treatment response: a replication. Biol. Psychiatry. https://doi.org/10.1016/s0006-3223(00)00619-3

Brody, A.L., Saxena, S., Mandelkern, M.A., Fairbanks, L.A., Ho, M.L., Baxter, L.R., 2001. Brain metabolic changes associated with symptom factor improvement in major depressive disorder. Biol. Psychiatry. https://doi.org/10.1016/S0006-3223(01)01117-9

Canli, T., Cooney, R.E., Goldin, P., Shah, M., Sivers, H., Thomason, M.E., Whitfield-Gabrieli, S., Gabriels, J.D.E., Gotlib, I.H., 2005. Amygdala reactivity to emotional faces predicts improvement in major depression. Neuroreport. https://doi.org/10.1097/01.wnr.0000174407.09515.cc

Chen, C.H., Ridler, K., Suckling, J., Williams, S., Fu, C.H.Y., Merlo-Pich, E., Bullmore, E., 2007. Brain Imaging Correlates of Depressive Symptom Severity and Predictors of Symptom Improvement After Antidepressant Treatment. Biol. Psychiatry. https://doi.org/10.1016/j.biopsych.2006.09.018

Chen, G., Saad, Z.S., Britton, J.C., Pine, D.S., Cox, R.W., 2013. Linear mixed-effects modeling approach to FMRI group analysis. Neuroimage 73, 176–190. https://doi.org/10.1016/J.NEUROIMAGE.2013.01.047

Costafreda, S.G., Khanna, A., Mourao-Miranda, J., Fu, C.H.Y., 2009. Neural correlates of sad faces predict clinical remission to cognitive behavioural therapy in depression. Neuroreport. https://doi.org/10.1097/WNR.0b013e3283294159

Cullen, K.R., Klimes-Dougan, B., Vu, D.P., Westlund Schreiner, M., Mueller, B.A., Eberly, L.E., Camchong, J., Westervelt, A., Lim, K.O., 2016. Neural Correlates of Antidepressant Treatment Response in Adolescents with Major Depressive Disorder. J. Child Adolesc. Psychopharmacol. https://doi.org/10.1089/cap.2015.0232

Davidson, R.J., Irwin, W., Anderle, M.J., Kalin, N.H., 2003. The neural substrates of affective processing in depressed patients treated with venlafaxine. Am. J. Psychiatry. https://doi.org/10.1176/appi.ajp.160.1.64

Delaveau, P., Jabourian, M., Lemogne, C., Allaïli, N., Choucha, W., Girault, N., Lehericy, S., Laredo, J., Fossati, P., 2016. Antidepressant short-term and long-term brain effects during self-referential processing in major depression. Psychiatry Res. - Neuroimaging. https://doi.org/10.1016/j.pscychresns.2015.11.007

Dichter, G.S., Felder, J.N., Smoski, M.J., 2010. The effects of Brief Behavioral Activation Therapy for Depression on cognitive control in affective contexts: An fMRI investigation. J. Affect. Disord. https://doi.org/10.1016/j.jad.2010.03.022

Doerig, N., Krieger, T., Altenstein, D., Schlumpf, Y., Spinelli, S., Späti, J., Brakowski, J., Quednow, B.B., Seifritz, E., Holtforth, M.G., 2016. Amygdala response to self-critical stimuli and symptom improvement in psychotherapy for depression. Br. J. Psychiatry. https://doi.org/10.1192/bjp.bp.114.149971

Fales, C.L., Barch, D.M., Rundle, M.M., Mintun, M.A., Mathews, J., Snyder, A.Z., Sheline, Y.I., 2009. Antidepressant treatment normalizes hypoactivity in dorsolateral prefrontal cortex during emotional interference processing in major depression. J. Affect. Disord. https://doi.org/10.1016/j.jad.2008.04.027

Fang, J., Egorova, N., Rong, P., Liu, J., Hong, Y., Fan, Y., Wang, X., Wang, H., Yu, Y., Ma, Y., Xu, C., Li, S., Zhao, J., Luo, M., Zhu, B., Kong, J., 2017. Early cortical biomarkers of longitudinal transcutaneous vagus nerve stimulation treatment success in depression. NeuroImage Clin. https://doi.org/10.1016/j.nicl.2016.12.016

Fitzgerald, P.B., Sritharan, A., Daskalakis, Z.J., De Castella, A.R., Kulkarni, J., Egan, G., 2007. A functional magnetic resonance imaging study of the effects of low frequency right prefrontal transcranial magnetic stimulation in depression. J. Clin. Psychopharmacol. https://doi.org/10.1097/jcp.0b013e318151521c

Forbes, E.E., Olino, T.M., Ryan, N.D., Birmaher, B., Axelson, D., Moyles, D.L., Dahl, R.E., 2010. Reward-related brain function as a predictor of treatment response in adolescents with major depressive disorder. Cogn. Affect. Behav. Neurosci. https://doi.org/10.3758/CABN.10.1.107

Frodl, T., Scheuerecker, J., Schoepf, V., Linn, J., Koutsouleris, N., Bokde, A.L.W., Hampel, H., Möller, H.J., Brückmann, H., Wiesmann, M., Meisenzahl, E., 2011. Different effects of mirtazapine and venlafaxine on brain activation: An open randomized controlled fMRI study. J. Clin. Psychiatry. https://doi.org/10.4088/JCP.09m05393blu

Fröhner, J.H., Teckentrup, V., Smolka, M.N., Kroemer, N.B., 2019. Addressing the reliability fallacy in fMRI: Similar group effects may arise from unreliable individual effects. Neuroimage. https://doi.org/10.1016/j.neuroimage.2019.03.053

Fu, C.H.Y., Costafreda, S.G., Sankar, A., Adams, T.M., Rasenick, M.M., Liu, P.,

Donati, R., Maglanoc, L.A., Horton, P., Marangell, L.B., 2015. Multimodal functional and structural neuroimaging investigation of major depressive disorder following treatment with duloxetine. BMC Psychiatry. https://doi.org/10.1186/s12888-015-0457-2

Fu, C.H.Y., Williams, S.C.R., Brammer, M.J., Suckling, J., Kim, J., Cleare, A.J., Walsh, N.D., Mitterschiffthaler, M.T., Andrew, C.M., Pich, E.M., Bullmore, E.T., 2007. Neural responses to happy facial expressions in major depression following antidepressant treatment. Am. J. Psychiatry. https://doi.org/10.1176/ajp.2007.164.4.599

Fu, C.H.Y., Williams, S.C.R., Cleare, A.J., Brammer, M.J., Walsh, N.D., Kim, J., Andrew, C.M., Pich, E.M., Williams, P.M., Reed, L.J., Mitterschiffthaler, M.T., Suckling, J., Bullmore, E.T., 2004. Attenuation of the neural response to sad faces in major depression by antidepressant treatment: A prospective, event-related functional magnetic resonance imaging study. Arch. Gen. Psychiatry. https://doi.org/10.1001/archpsyc.61.9.877

Fu, C.H.Y., Williams, S.C.R., Cleare, A.J., Scott, J., Mitterschiffthaler, M.T., Walsh, N.D., Donaldson, C., Suckling, J., Andrew, C., Steiner, H., Murray, R.M., 2008. Neural Responses to Sad Facial Expressions in Major Depression Following Cognitive Behavioral Therapy. Biol. Psychiatry. https://doi.org/10.1016/j.biopsych.2008.04.033

Furey, M.L., Drevets, W.C., Hoffman, E.M., Frankel, E., Speer, A.M., Zarate, C.A., 2013. Potential of pretreatment neural activity in the visual cortex during emotional processing to predict treatment response to scopolamine in major depressive disorder. JAMA Psychiatry. https://doi.org/10.1001/2013.jamapsychiatry.60

Furey, M.L., Drevets, W.C., Szczepanik, J., Khanna, A., Nugent, A., Zarate, C.A., 2015. Pretreatment differences in BOLD response to emotional faces correlate with Antidepress ant response to scopolamine. Int. J. Neuropsychopharmacol. https://doi.org/10.1093/ijnp/pyv028

Godlewska, B.R., Browning, M., Norbury, R., Cowen, P.J., Harmer, C.J., 2016. Early changes in emotional processing as a marker of clinical response to SSRI treatment in depression. Transl. Psychiatry. https://doi.org/10.1038/tp.2016.130

Godlewska, B.R., Browning, M., Norbury, R., Igoumenou, A., Cowen, P.J., Harmer, C.J., 2018. Predicting Treatment Response in Depression: The Role of Anterior Cingulate Cortex. Int. J. Neuropsychopharmacol. https://doi.org/10.1093/ijnp/pyy069

Godlewska, B.R., Norbury, R., Selvaraj, S., Cowen, P.J., Harmer, C.J., 2012. Short-term SSRI treatment normalises amygdala hyperactivity in depressed patients. Psychol. Med. https://doi.org/10.1017/S0033291712000591

Gyurak, A., Patenaude, B., Korgaonkar, M.S., Grieve, S.M., Williams, L.M., Etkin, A., 2016. Frontoparietal activation during response inhibition predicts remission to antidepressants in patients with major depression. Biol. Psychiatry.

https://doi.org/10.1016/j.biopsych.2015.02.037

Hamilton, J.P., Glover, G.H., Bagarinao, E., Chang, C., Mackey, S., Sacchet, M.D., Gotlib, I.H., 2016. Effects of salience-network-node neurofeedback training on affective biases in major depressive disorder. Psychiatry Res. - Neuroimaging. https://doi.org/10.1016/j.pscychresns.2016.01.016

Harmonya, T., Fernández, T., Rodríguez, M., Reyes, A., Marosi, E., Bernal, J., 1993. Test-retest reliability of EEG spectral parameters during cognitive tasks: II coherence. Int. J. Neurosci. https://doi.org/10.3109/00207459308994281

Heller, A.S., Johnstone, T., Light, S.N., Peterson, M.J., Kolden, G.G., Kalin, N.H., Davidson, R.J., 2013. Relationships between changes in sustained fronto-striatal connectivity and positive affect in major depression resulting from antidepressant treatment. Am. J. Psychiatry. https://doi.org/10.1176/appi.ajp.2012.12010014

Keedwell, P.A., Drapier, D., Surguladze, S., Giampietro, V., Brammer, M., Phillips, M., 2010. Subgenual cingulate and visual cortex responses to sad faces predict clinical outcome during antidepressant treatment for depression. J. Affect. Disord. https://doi.org/10.1016/j.jad.2009.04.031

Langenecker, S.A., Kennedy, S.E., Guidotti, L.M., Briceno, E.M., Own, L.S., Hooven, T., Young, E.A., Akil, H., Noll, D.C., Zubieta, J.K., 2007. Frontal and Limbic Activation During Inhibitory Control Predicts Treatment Response in Major Depressive Disorder. Biol. Psychiatry. https://doi.org/10.1016/j.biopsych.2007.02.019

Lemogne, C., Mayberg, H., Bergouignan, L., Volle, E., Delaveau, P., Lehéricy, S., Allilaire, J.F., Fossati, P., 2010. Self-referential processing and the prefrontal cortex over the course of depression: A pilot study. J. Affect. Disord. https://doi.org/10.1016/j.jad.2009.11.003

Light, S.N., Heller, A.S., Johnstone, T., Kolden, G.G., Peterson, M.J., Kalin, N.H., Davidson, R.J., 2011. Reduced right ventrolateral prefrontal cortex activity while inhibiting positive affect is associated with improvement in hedonic capacity after 8 weeks of antidepressant treatment in major depressive disorder. Biol. Psychiatry. https://doi.org/10.1016/j.biopsych.2011.06.031

Linden, D.E.J., Habes, I., Johnston, S.J., Linden, S., Tatineni, R., Subramanian, L., Sorger, B., Healy, D., Goebel, R., 2012. Real-time self-regulation of emotion networks in patients with depression. PLoS One. https://doi.org/10.1371/journal.pone.0038115

López-Solà, M., Pujol, J., Hernández-Ribas, R., Harrison, B.J., Contreras-Rodríguez, O., Soriano-Mas, C., Deus, J., Ortiz, H., Menchón, J.M., Vallejo, J., Cardoner, N., 2010. Effects of duloxetine treatment on brain response to painful stimulation in major depressive disorder. Neuropsychopharmacology. https://doi.org/10.1038/npp.2010.108

MacDuffie, K.E., MacInnes, J., Dickerson, K.C., Eddington, K.M., Strauman, T.J., Adcock, R.A., 2018. Single session real-time fMRI neurofeedback has a lasting impact on cognitive behavioral therapy strategies. NeuroImage Clin.

https://doi.org/10.1016/j.nicl.2018.06.009

Mayberg, H.S., Brannan, S.K., Mahurin, R.K., Jerabek, P.A., Brickman, J.S., Tekell, J.L., Silva, J.A., McGinnis, S., Glass, T.G., Martin, C.C., Fox, P.T., 1997. Cingulate function in depression: A potential predictor of treatment response. Neuroreport. https://doi.org/10.1097/00001756-199703030-00048

McEvoy, L.K., Smith, M.E., Gevins, A., 2000. Test-retest reliability of cognitive EEG. Clin. Neurophysiol. https://doi.org/10.1016/S1388-2457(99)00258-8

Mehler, D.M.A., Sokunbi, M.O., Habes, I., Barawi, K., Subramanian, L., Range, M., Evans, J., Hood, K., Lührs, M., Keedwell, P., Goebel, R., Linden, D.E.J., 2018. Targeting the affective brain—a randomized controlled trial of real-time fMRI neurofeedback in patients with depression. Neuropsychopharmacology. https://doi.org/10.1038/s41386-018-0126-5

Miller, J.M., Schneck, N., Siegle, G.J., Chen, Y., Ogden, R.T., Kikuchi, T., Oquendo, M.A., Mann, J.J., Parsey, R. V., 2013. FMRI response to negative words and SSRI treatment outcome in major depressive disorder: A preliminary study. Psychiatry Res. - Neuroimaging. https://doi.org/10.1016/j.pscychresns.2013.08.001

Nitschke, J.B., Sarinopoulos, I., Oathes, D.J., Johnstone, T., Whalen, P.J., Davidson, R.J., Kalin, N.H., 2009. Anticipatory activation in the Amygdala and Anterior Cingulate in generalized anxiety disorder and prediction of reatment response. Am. J. Psychiatry. https://doi.org/10.1176/appi.ajp.2008.07101682

Opmeer, E.M., Kortekaas, R., Van Tol, M.J., Renken, R.J., Demenescu, L.R., Woudstra, S., Ter Horst, G.J., Van Buchem, M.A., Van Der Wee, N.J.A., Veltman, D.J., Aleman, A., 2016. Changes in regional brain activation related to depressive state: A 2-year longitudinal functional MRI study. Depress. Anxiety. https://doi.org/10.1002/da.22425

Pizzagalli, D., Pascual-Marqui, R.D., Nitschke, J.B., Oakes, T.R., Larson, C.L., Abercrombie, H.C., Schaefer, S.M., Koger, J. V., Benca, R.M., Davidson, R.J., 2001. Anterior cingulate activity as a predictor of degree of treatment response in major depression: Evidence from brain electrical tomography analysis. Am. J. Psychiatry. https://doi.org/10.1176/appi.ajp.158.3.405

Pollock, V.E., Schneider, L.S., Lyness, S.A., 1991. Reliability of topographic quantitative EEG amplitude in healthy late-middle-aged and elderly subjects. Electroencephalogr. Clin. Neurophysiol. https://doi.org/10.1016/0013-4694(91)90152-T

Ritchey, M., Dolcos, F., Eddington, K.M., Strauman, T.J., Cabeza, R., 2011. Neural correlates of emotional processing in depression: Changes with cognitive behavioral therapy and predictors of treatment response. J. Psychiatr. Res. https://doi.org/10.1016/j.jpsychires.2010.09.007

Rizvi, S.J., Salomons, T. V., Konarski, J.Z., Downar, J., Giacobbe, P., McIntyre, R.S., Kennedy, S.H., 2013. Neural response to emotional stimuli associated with successful antidepressant treatment and behavioral activation. J. Affect. Disord.

https://doi.org/10.1016/j.jad.2013.06.050

Robertson, B., Wang, L., Diaz, M.T., Aiello, M., Gersing, K., Beyer, J., Mukundan, S., McCarthy, G., Doraiswamy, P.M., 2007. Effect of bupropion extended release on negative emotion processing in major depressive disorder: A pilot functional magnetic resonance imaging study. J. Clin. Psychiatry. https://doi.org/10.4088/JCP.v68n0212

Rosenblau, G., Sterzer, P., Stoy, M., Park, S., Friedel, E., Heinz, A., Pilhatsch, M., Bauer, M., Ströhle, A., 2012. Functional neuroanatomy of emotion processing in major depressive disorder is altered after successful antidepressant therapy. J. Psychopharmacol. https://doi.org/10.1177/0269881112450779

Roy, M., Harvey, P.O., Berlim, M.T., Mamdani, F., Beaulieu, M.M., Turecki, G., Lepage, M., 2010. Medial prefrontal cortex activity during memory encoding of pictures and its relation to symptomatic improvement after citalopram treatment in patients with major depression. J. Psychiatry Neurosci. https://doi.org/10.1503/jpn.090010

Rubin-Falcone, H., Weber, J., Kishon, R., Ochsner, K., Delaparte, L., Doré, B., Zanderigo, F., Oquendo, M.A., Mann, J.J., Miller, J.M., 2018. Longitudinal effects of cognitive behavioral therapy for depression on the neural correlates of emotion regulation. Psychiatry Res. - Neuroimaging. https://doi.org/10.1016/j.pscychresns.2017.11.002

Ruhé, H.G., Booij, J., Veltman, D.J., Michel, M.C., Schene, A.H., 2012. Successful pharmacologic treatment of major depressive disorder attenuates amygdala activation to negative facial expressions: A functional magnetic resonance imaging study. J. Clin. Psychiatry. https://doi.org/10.4088/JCP.10m06584

Salinsky, M.C., Oken, B.S., Morehead, L., 1991. Test-retest reliability in EEG frequency analysis. Electroencephalogr. Clin. Neurophysiol. https://doi.org/10.1016/0013-4694(91)90203-G

Samson, A.C., Meisenzahl, E., Scheuerecker, J., Rose, E., Schoepf, V., Wiesmann, M., Frodl, T., 2011. Brain activation predicts treatment improvement in patients with major depressive disorder. J. Psychiatr. Res. https://doi.org/10.1016/j.jpsychires.2011.03.009

Sankar, A., Adams, T.M., Costafreda, S.G., Marangell, L.B., Fu, C.H.Y., 2017. Effects of antidepressant therapy on neural components of verbal working memory in depression. J. Psychopharmacol. https://doi.org/10.1177/0269881117724594

Schaefer, H.S., Putnam, K.M., Benca, R.M., Davidson, R.J., 2006. Event-Related Functional Magnetic Resonance Imaging Measures of Neural Activity to Positive Social Stimuli in Pre- and Post-Treatment Depression. Biol. Psychiatry. https://doi.org/10.1016/j.biopsych.2006.03.024

Sheline, Y.I., Barch, D.M., Donnelly, J.M., Ollinger, J.M., Snyder, A.Z., Mintun, M.A., 2001. Increased amygdala response to masked emotional faces in depressed subjects resolves with antidepressant treatment: An fMRI study. Biol. Psychiatry.

https://doi.org/10.1016/S0006-3223(01)01263-X

Shrout, P.E., Fleiss, J.L., 1979. Intraclass correlations: Uses in assessing rater reliability. Psychol. Bull. https://doi.org/10.1037/0033-2909.86.2.420

Siegle, G.J., Carter, C.S., Thase, M.E., 2006. Use of fMRI to predict recovery from unipolar depression with cognitive behavior therapy. Am. J. Psychiatry. https://doi.org/10.1176/ajp.2006.163.4.735

Siegle, G.J., Thompson, W., Carter, C.S., Steinhauer, S.R., Thase, M.E., 2007. Increased Amygdala and Decreased Dorsolateral Prefrontal BOLD Responses in Unipolar Depression: Related and Independent Features. Biol. Psychiatry. https://doi.org/10.1016/j.biopsych.2006.05.048

Siegle, G.J., Thompson, W.K., Collier, A., Berman, S.R., Feldmiller, J., Thase, M.E., Friedman, E.S., 2012. Toward Clinically Useful Neuroimaging in Depression Treatment. Arch. Gen. Psychiatry. https://doi.org/10.1001/archgenpsychiatry.2012.65

Spies, M., Kraus, C., Geissberger, N., Auer, B., Klöbl, M., Tik, M., Störkat, I.L., Hahn, A., Woletz, M., Pfabigan, D.M., Kasper, S., Lamm, C., Windischberger, C., Lanzenberger, R., 2017. Default mode network deactivation during emotion processing predicts early antidepressant response. Transl. Psychiatry. https://doi.org/10.1038/tp.2016.265

Stoy, M., Schlagenhauf, F., Sterzer, P., Bermpohl, F., Hägele, C., Suchotzki, K., Schmack, K., Wrase, J., Ricken, R., Knutson, B., Adli, M., Bauer, M., Heinz, A., Ströhle, A., 2012. Hyporeactivity of ventral striatum towards incentive stimuli in unmedicated depressed patients normalizes after treatment with escitalopram. J. Psychopharmacol. https://doi.org/10.1177/0269881111416686

Straub, J., Plener, P.L., Sproeber, N., Sprenger, L., Koelch, M.G., Groen, G., Abler, B., 2015. Neural correlates of successful psychotherapy of depression in adolescents. J. Affect. Disord. https://doi.org/10.1016/j.jad.2015.05.020

Strege, M. V, Siegle, G.J., Young, K., 2020. Cingulate prediction of response to antidepressant and cognitive behavioral therapies for depression: Theory, meta-analysis, and empirical application. bioRxiv 2020.12.02.407841. https://doi.org/10.1101/2020.12.02.407841

Szczepanik, J., Nugent, A.C., Drevets, W.C., Khanna, A., Zarate, C.A., Furey, M.L., 2016. Amygdala response to explicit sad face stimuli at baseline predicts antidepressant treatment response to scopolamine in major depressive disorder. Psychiatry Res. - Neuroimaging. https://doi.org/10.1016/j.pscychresns.2016.06.005

Tao, R., Calley, C.S., Hart, J., Mayes, T.L., Nakonezny, P.A., Lu, H., Kennard, B.D., Tamminga, C.A., Emslie, G.J., 2012. Brain activity in adolescent major depressive disorder before and after fluoxetine treatment. Am. J. Psychiatry. https://doi.org/10.1176/appi.ajp.2011.11040615

Toki, S., Okamoto, Y., Onoda, K., Matsumoto, T., Yoshimura, S., Kunisato, Y.,

Okada, G., Shishida, K., Kobayakawa, M., Fukumoto, T., Machino, A., Inagaki, M., Yamawaki, S., 2014. Hippocampal activation during associative encoding of word pairs and its relation to symptomatic improvement in depression: A functional and volumetric MRI study. J. Affect. Disord. https://doi.org/10.1016/j.jad.2013.07.021

Victor, T.A., Furey, M.L., Fromm, S.J., Öhman, A., Drevets, W.C., 2013. Changes in the neural correlates of implicit emotional face processing during antidepressant treatment in major depressive disorder, in: International Journal of Neuropsychopharmacology. https://doi.org/10.1017/S146114571300062X

Victor, T.A., Furey, M.L., Fromm, S.J., Öhman, A., Drevets, W.C., 2010. Relationship between amygdala responses to masked faces and mood state and treatment in major depressive disorder. Arch. Gen. Psychiatry. https://doi.org/10.1001/archgenpsychiatry.2010.144

Wagner, G., Koch, K., Schachtzabel, C., Sobanski, T., Reichenbach, J.R., Sauer, H., Schlösser, R.G.M., 2010. Differential effects of serotonergic and noradrenergic antidepressants on brain activity during a cognitive control task and neurofunctional prediction of treatment outcome in patients with depression. J. Psychiatry Neurosci. https://doi.org/10.1503/jpn.090081

Walsh, N.D., Williams, S.C.R., Brammer, M.J., Bullmore, E.T., Kim, J., Suckling, J., Mitterschiffthaler, M.T., Cleare, A.J., Pich, E.M., Mehta, M.A., Fu, C.H.Y., 2007. A Longitudinal Functional Magnetic Resonance Imaging Study of Verbal Working Memory in Depression After Antidepressant Therapy. Biol. Psychiatry. https://doi.org/10.1016/j.biopsych.2006.12.022

Wang, Y., Xu, C., Cao, X., Gao, Q., Li, J., Liu, Z., Sun, N., Ren, Y., Zhang, K., 2012. Effects of an antidepressant on neural correlates of emotional processing in patients with major depression. Neurosci. Lett. https://doi.org/10.1016/j.neulet.2012.08.034

Williams, L.M., Korgaonkar, M.S., Song, Y.C., Paton, R., Eagles, S., Goldstein-Piekarski, A., Grieve, S.M., Harris, A.W.F., Usherwood, T., Etkin, A., 2015. Amygdala Reactivity to Emotional Faces in the Prediction of General and Medication-Specific Responses to Antidepressant Treatment in the Randomized iSPOT-D Trial. Neuropsychopharmacology. https://doi.org/10.1038/npp.2015.89

Yoshimura, S., Okamoto, Y., Onoda, K., Matsunaga, M., Okada, G., Kunisato, Y., Yoshino, A., Ueda, K., Suzuki, S. ichi, Yamawaki, S., 2014. Cognitive behavioral therapy for depression changes medial prefrontal and ventral anterior cingulate cortex activity associated with self-referential processing. Soc. Cogn. Affect. Neurosci. https://doi.org/10.1093/scan/nst009

Young, K.D., Misaki, M., Harmer, C.J., Victor, T., Zotev, V., Phillips, R., Siegle, G.J., Drevets, W.C., Bodurka, J., 2017a. Real-Time fMRI Amygdala Neurofeedback Changes Positive Information Processing in Major Depressive Disorder. Biol. Psychiatry. https://doi.org/10.1016/j.biopsych.2017.03.013

Young, K.D., Siegle, G.J., Misaki, M., Zotev, V., Phillips, R., Drevets, W.C., Bodurka,

J., 2018. Altered task-based and resting-state amygdala functional connectivity following real-time fMRI amygdala neurofeedback training in major depressive disorder. NeuroImage Clin. https://doi.org/10.1016/j.nicl.2017.12.004

Young, K.D., Siegle, G.J., Zotev, V., Phillips, R., Misaki, M., Yuan, H., Drevets, W.C., Bodurka, J., 2017b. Randomized clinical trial of real-time fMRI amygdala neurofeedbackfor major depressive disorder: Effectson symptoms and autobiographical memory recall, in: American Journal of Psychiatry. https://doi.org/10.1176/appi.ajp.2017.16060637

Young, K.D., Zotev, V., Phillips, R., Misaki, M., Yuan, H., Drevets, W.C., Bodurka, J., 2014. Real-time fMRI neurofeedback training of amygdala activity in patients with major depressive disorder. PLoS One. https://doi.org/10.1371/journal.pone.0088785

Yuan, H., Young, K.D., Phillips, R., Zotev, V., Misaki, M., Bodurka, J., 2014. Resting-State Functional Connectivity Modulation and Sustained Changes After Real-Time Functional Magnetic Resonance Imaging Neurofeedback Training in Depression. Brain Connect. https://doi.org/10.1089/brain.2014.0262

Zotev, V., Phillips, R., Yuan, H., Misaki, M., Bodurka, J., 2014. Self-regulation of human brain activity using simultaneous real-time fMRI and EEG neurofeedback. Neuroimage. https://doi.org/10.1016/j.neuroimage.2013.04.126

Zotev, V., Yuan, H., Misaki, M., Phillips, R., Young, K.D., Feldner, M.T., Bodurka, J., 2016. Correlation between amygdala BOLD activity and frontal EEG asymmetry during real-time fMRI neurofeedback training in patients with depression. NeuroImage Clin. https://doi.org/10.1016/j.nicl.2016.02.003
